# SPCS1-Dependent E2-p7 processing determines HCV Assembly efficiency

**DOI:** 10.1371/journal.ppat.1010310

**Published:** 2022-02-07

**Authors:** Nabeel Alzahrani, Ming-Jhan Wu, Carla F. Sousa, Olga V. Kalinina, Christoph Welsch, MinKyung Yi

**Affiliations:** 1 Department of Microbiology and Immunology, University of Texas Medical Branch at Galveston, Galveston, Texas, United States of America; 2 Drug Bioinformatics Group, HIPS, HZI, Saarbrücken, Germany; 3 Medical Faculty, Saarland University, Homburg, Germany; 4 Department of Internal Medicine 1, Goethe University Hospital, Frankfurt am Main, Germany; University of California, San Diego, UNITED STATES

## Abstract

Recent studies identified signal peptidase complex subunit 1 (SPCS1) as a proviral host factor for Flaviviridae viruses, including HCV. One of the SPCS1’s roles in flavivirus propagation was attributed to its regulation of signal peptidase complex (SPC)-mediated processing of flavivirus polyprotein, especially C-prM junction. However, whether SPCS1 also regulates any SPC-mediated processing sites within HCV polyprotein remains unclear. In this study, we determined that loss of SPCS1 specifically impairs the HCV E2-p7 processing by the SPC. We also determined that efficient separation of E2 and p7, regardless of its dependence on SPC-mediated processing, leads to SPCS1 dispensable for HCV assembly These results suggest that SPCS1 regulates HCV assembly by facilitating the SPC-mediated processing of E2-p7 precursor. Structural modeling suggests that intrinsically delayed processing of the E2-p7 is likely caused by the structural rigidity of p7 N-terminal transmembrane helix-1 (p7/TM1/helix-1), which has mostly maintained membrane-embedded conformations during molecular dynamics (MD) simulations. E2-p7-processing-impairing p7 mutations narrowed the p7/TM1/helix-1 bending angle against the membrane, resulting in closer membrane embedment of the p7/TM1/helix-1 and less access of E2-p7 junction substrate to the catalytic site of the SPC, located well above the membrane in the ER lumen. Based on these results we propose that the key mechanism of action of SPCS1 in HCV assembly is to facilitate the E2-p7 processing by enhancing the E2-p7 junction site presentation to the SPC active site. By providing evidence that SPCS1 facilitates HCV assembly by regulating SPC-mediated cleavage of E2-p7 junction, equivalent to the previously established role of this protein in C-prM junction processing in flavivirus, this study establishes the common role of SPCS1 in Flaviviridae family virus propagation as to exquisitely regulate the SPC-mediated processing of specific, suboptimal target sites.

## Author summary

Host factor SPCS1 plays a critical role in the propagation of Flaviviridae viruses, including HCV. However, it remained unclear whether the mechanism of action (s) of SPCS1 for these different viral genera is similar or different. We elucidated that SPCS1 facilitates HCV assembly by enhancing the intrinsically suboptimal, SPC-mediated processing of the HCV E2-p7 precursor. By performing in silico analyses, we also found that the N-terminal domain of the p7, succeeding the cleavage junction, maintains a rigid angle relative to the membrane z-axis, potentially hiding the E2-p7 junction site within the membrane and out of reach from the SPC catalytic site, providing an explanation for the suboptimal processing of this junction. Enhancing the E2 and p7 separation in either SPC-dependent or independent manner, made SPCS1 dispensable for HCV assembly. Based on these results we propose that SPCS1 facilitates HCV assembly by enhancing E2-p7 junction substrate presentation to SPC-mediated proteolysis. A previous study showed that flavivirus propagation depends on SPCS1-mediated facilitation of SPC-mediated processing of C-prM precursor, which also shows the SPC cleavage unfavorable substrate feature. Therefore, our results establish that SPCS1 plays a common role in Flaviviridae family virus propagation, which is to facilitate the SPC-mediated processing of specific, suboptimal target sites.

## Introduction

Hepatitis C virus (HCV) is the main causative agent of severe liver diseases, including chronic hepatitis, cirrhosis, and hepatocellular carcinoma [1]. According to the World Health Organization (WHO) report, about 58 million people worldwide are estimated to have chronic HCV infection, leading to 290,000 annual deaths. Despite the availability of highly effective direct-acting antivirals that could cure >90% of HCV infections [2,3], HCV elimination is challenged by continuing new infections (1.5 million per year according to WHO estimate), under-diagnosis, and under-treatment issues [4].

HCV is a positive-stranded RNA virus belonging to the Hepacivirus genus in the Flaviviridae family. It encodes an open reading frame for a single polyprotein flanked by 5’- and 3’- non-coding regions. Host signal peptidase complex (SPC) processes the N-terminal region of HCV polyprotein encoding the structural proteins (Core, E1, and E2) and the two accessory proteins (p7 and NS2) [5,6]. The Core protein is further processed by signal peptide peptidase complexes [7,8]. The NS2 cysteine protease mediates autocleavage of NS2-NS3 junction, facilitated by the N-terminal region of NS3 [9,10]. The NS3-NS4A-NS4B-NS5A-NS5B polyprotein is processed by NS3 serine protease together with its co-factor NS4A leading to replicase complex formation involved in viral RNA replication [11,12].

With some exceptions, SPC-mediated processing of signal peptide from the nascent polypeptide chain occurs co-translationally [13–15]. Consistent with this general rule, most of the SPC-mediated cleavages of HCV polyprotein precursor occurs during or shortly after translation [5,6,16]. The exception to this rule is the processing of E2-p7, and to a lesser extent, p7-NS2 junction sites, which are delayed and incomplete [6,17–19]. E2-p7 junction cleavage seems to occur post-translationally since the bulk of E2-p7 processing occurred during the chase period lasting 6 hr or more in the pulse-chase analysis [20,21]. The study by Carrère-Kremer *et al*. suggested that the structural constraints surrounding the p7 junction sites likely caused the inefficient cleavage of these junction sites [17]. Importantly, delayed, suboptimal processing of the E2-p7 junction site is critical for HCV assembly, since promoting the separation of E2 and p7 by introducing the mutations, epitope tags or EMCV IRES around the E2-p7 junction inhibited HCV assembly [18,22,23]. On the other hand, mutations that severely impaired E2-p7 processing resulted in complete inhibition of HCV assembly [18,23]. These results suggest that efficient HCV assembly depends on the exquisite regulation of E2-p7 processing. However, other than the contribution by structural determinants in the p7 region as described above, the regulatory mechanism involved in the E2-p7 processing remains incompletely defined.

The eukaryotic SPC is composed of five subunits including two isoforms of catalytic subunits [SEC11A (SPC18) and SEC11C (SPC21)] and three regulatory subunits including signal peptidase complex subunit 1 [SPCS1 (SPC12)], SPCS2 (SPC25), and SPCS3 (SPC22/23) [24–30]. Among the SPC subunits, SEC11 and SPCS3 are essential for signal peptidase activity and cell survival [25,26,30]. SPCS2 interacts with the b subunit of Sec61 translocon complex likely facilitating co-translational signal peptidase processing [31,32] and, at high-temperature conditions, regulates the catalytic activity of the SPC and viability of yeast cells [27]. However, the role of SPCS1 has been less clear. SPCS1 knockout minimally affected signal peptidase processing and secretion of host proteins, as well as the survival of human cell lines [33]. Similarly, the yeast homolog of SPCS1 (Spc1p) was nonessential for signal peptidase activity and cell survival [28]. Interestingly, cleavage of the signal peptide from an ER-resident protein was inhibited in SPCS1(-) yeast cells upon overexpression of abnormal membrane protein indicating that SPCS1 could facilitate SPC-mediated cleavage under certain conditions [28]. On the other hand, the processing of signal anchored or model transmembrane proteins was enhanced in SPCS1(-) yeast cells proportional to increased length and hydrophobicity of the signal sequence suggesting that SPCS1 may negatively regulate the signal sequence cleavage by the SPC under other conditions [34]. These results imply that SPCS1 could either up or down-regulate SPC-mediated processing of selected substrates. However, the exact mechanism of action of SPCS1 remains incompletely defined.

Previously, by performing genome-wide siRNA screening, Li *et al*. identified the SPCS1, SPCS2, and SPCS3 as the proviral host factors involved in the late step of HCV replication [35]. Subsequently, Suzuki *et al*. determined that the SPCS1 is an NS2 interactor using a split-ubiquitin membrane yeast two-hybrid assay [36]. They further determined that SPCS1 participates in HCV assembly by interacting with E2 and NS2 without affecting HCV entry and viral RNA replication. Interestingly, SPCS1 was also shown to interact with Japanese encephalitis virus (JEV) NS2B, thereby facilitating its assembly [37]. These results suggest that SPCS1 enhances HCV and flavivirus assembly by interacting with different viral proteins.

Using CRISPR/Cas9-based screen, Zhang *et al*. also identified SPCS1 and SPCS3 as the proviral host factors for Flaviviridae family viruses, including flavivirus and HCV [33]. According to this report, loss of SPCS1 specifically impaired the SPC-mediated cleavage of the flavivirus C-prM junction site resulting in a marked reduction in flavivirus yield [33]. They also showed that lack of SPCS1 had little impact on the production of alpha viruses, bunyaviruses, rhabdoviruses, and the surface expression or secretion of diverse host proteins, despite that all these processes require SPC-mediated signal sequence processing [33]. These results support the target exclusivity of SPCS1 action during SPC-mediated processing [28, 34].

The processing at HCV E2-p7 and flavivirus C-prM junction sites share similar phenotypic consequences [19]. First, SPC-medicated cleavage at either of these substrate sites was a posttranslational, delayed event [17–19,38,39]. Second, enhancing or further inhibiting cleavage at these sites led to inhibition of virus assembly [22,40,41]. Since SPCS1’s role in flavivirus assembly depends on its regulation of C-prM junction cleavage, we hypothesize that SPCS1’s role in HCV assembly also depends on its regulation of E2-p7 cleavage. To test this hypothesis, we investigated two specific questions: First, does HCV also exploit the SPCS1 to regulate SPC-mediated E2-p7 processing? Second, does the SPCS1’s role in HCV assembly depend on the SPC-mediated processing of the E2-p7 junction site? Our results indicate that SPCS1 does facilitate E2-p7 processing. In addition, enhancing E2 and p7 separation, regardless of SPC involvement in this process, leads to SPCS1 dispensable for HCV assembly. We believe that, with this work, we established that HCV and flaviviruses, belonging to the same Flaviviridae family, similarly exploit the function of host factor SPCS1 to exquisitely regulate SPC-mediated cleavage of viral protein precursors to facilitate virus assembly.

## Results

### SPCS1 facilitates HCV E2-p7 processing regardless of HCV genotypes

**Fig 1:**
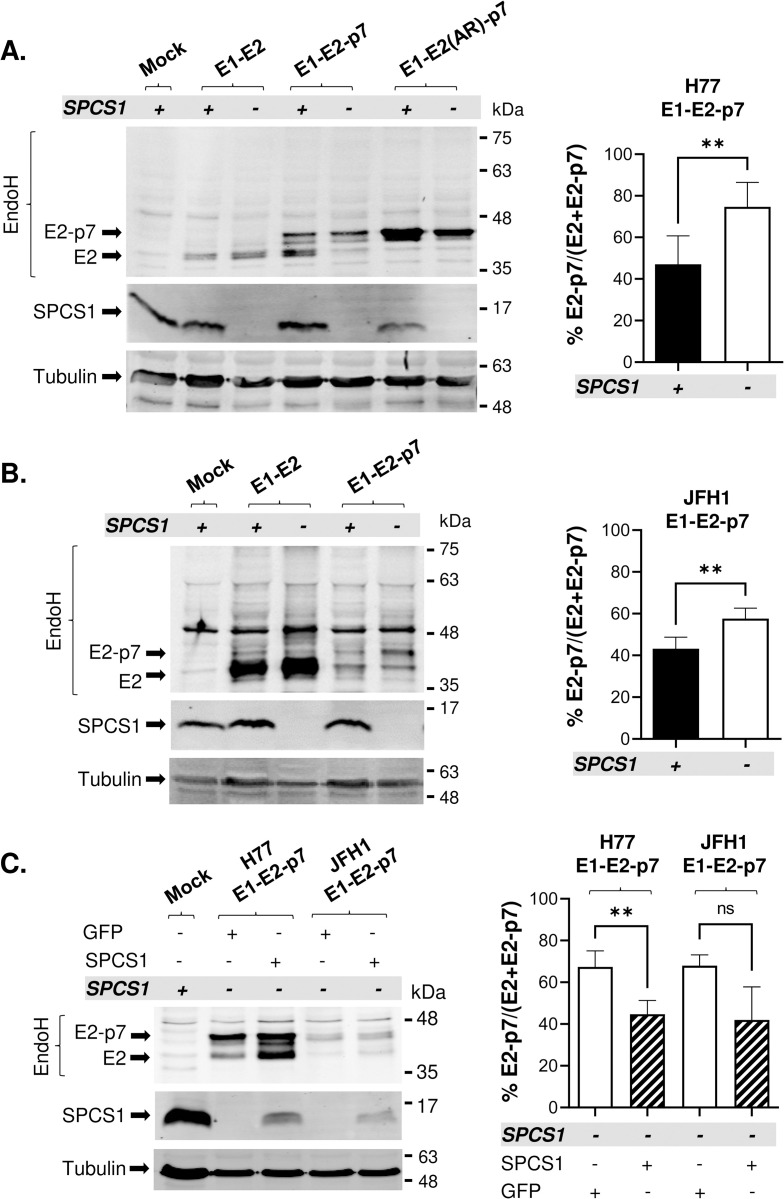
The effect of SPCS1 loss on HCV E2-p7 processing in an ectopic protein expression system. Detection of E2 derivatives and SPCS1 by western blot following transfection of plasmids encoding either E1-E2, E1-E2-p7, or E1-E2(AR)-p7 sequences from **(A)** HCV gt1a (H77) or **(B)** gt2a (JFH1) to SPCS1(-) and SPCS1(+) Huh 7.5 cells. H77 E2 and E2-p7 are detected in doublet bands upon Endo H treatment due to incomplete deglycosylation, as described before [18, 86–88]. **(C)** The effect of SPCS1 trans-complementation in SPCS1(-) cells on E2-p7 processing. The SPCS1(-) were co-transfected with H77 or JFH1 E1-E2-p7 and GFP or SPCS1-encoding plasmids followed by western blot analysis using anti-E2 or anti-SPCS1 antibodies. E2 western blot was performed following Endo H treatment to better separate E2 and E2-p7. The mean percentage of E2-p7 precursor and the standard deviation shown at the right are generated using results from three to five biological replicates of representative western blot data shown at the left. Mock represents non-transfected control SPCS1(+) cells. Statistical analyses were performed using GraphPad Prism, version 9, software. Asterisks indicate statistically significant differences between two paired values: **, P < 0.005. The differences with a P-value of >0.05 were considered not significant (ns).

We determined the role of SPCS1 in HCV E2-p7 cleavage by comparing the relative levels of unprocessed E2-p7 among total E2 (E2 + E2-p7) in control [SPCS1(+)] and SPCS1 knock-out [SPCS1(-)] Huh-7.5 cells [33]. We began our study by using genotype (gt) 1a H77 strain-derived E2 precursors, including E1-E2, E1-E2-p7, and E1-E2 (AR)-p7 (defective in E2-p7 cleavage due to Alanine to Arginine mutation at the p1 position of the SPC cleavage site) [18]. To assess the role of SPCS1 on SPC-mediated processing of these precursors, plasmids encoding E2 derivatives were expressed in SPCS1(+) and SPCS1(-) cells. The cell lysates were pretreated with Endoglycosidase H (Endo H) to better separate E2 and E2-p7 before SDS-polyacrylamide gel electrophoresis [18]. Then, the relative efficiency of E2-p7 precursor processing was assessed by performing western blot analysis using an anti-E2 antibody. The locations of E2 and E2-p7 on the western blot membrane were deduced from those of E2 and E2(AR)-p7 processed from E1-E2 and E1-E2(AR)-p7, respectively, (Fig 1A, left panel). Regardless of SPCS1 expression, we did not detect anti-E2 antibody detectable protein bands corresponding to the expected sizes of E1-E2 precursors (about 58 kDa and 65 kDa for deglycosylated E1-E2 and E1-E2-p7 respectively) from any of the cell lysates expressing E1-E2 encoding precursors described above (Fig 1A). These results suggest that E1-E2 processing occurred efficiently and SPCS1 has little impact on SPC-mediated H77 E1-E2 processing (Fig 1A, left panel). On the other hand, loss of SPCS1 significantly increased the the percentage of E2-p7 level relative to the total level of E2 (E2+E2-p7) (Fig 1A). These results suggest that SPCS1 specifically facilitates SPC-mediated H77 E2-p7 processing.

Although the study by Suzuki *et al*. concluded that SPCS1 does not impact the processing of gt2a JFH1 structural proteins [36], this study did not provide the data evaluating the SPCS1’s role on JFH1 E2-p7 processing. To determine whether SPCS1 plays a role in HCV E2-p7 processing in different HCV genotypes, we assessed the effect of SPCS1 in JFH1 E2-p7 processing. In western blot analysis, while JFH1 E2 from E1-E2 precursor was readily detectable by anti-E2 antibody, that from E1-E2-p7 precursor, including E2 and E2-p7, detectable by the same antibody was reduced (Fig 1B, left panel). This phenotype may be due to the weak recognition of Endo H treated JFH1 E2-p7 precursor by the anti-E2 antibody used in this study, rather than instability of JFH1 E2-p7 precursor, since proteasome inhibitor treatment did not alter the JFH1 E2-p7 level in western blot analysis (S2B Fig). Nonetheless, consistent with the results from gt1a H77-derived proteins, loss of SPCS1 reduced JFH1 E2-p7 processing without affecting E1-E2 processing, as evidenced by lack of anti-E2 detectable protein bands at the expected sizes of E1-E2 precursors (Fig 1B). These results demonstrated that SPCS1 specifically facilitates both gt1a and gt2a HCV E2-p7 processing mediated by SPC.

To make sure that the reduced E2-p7 processing in the SPCS1(-) cells is indeed caused by the lack of SPCS1, we conducted the rescue experiments by co-transfecting E1-E2-p7 encoding plasmid along with GFP or SPCS1 expressing plasmids into SPCS1(-) cells. Compared to co-expression of GFP, that of SPCS1 consistently reduced the relative percentages of H77- or JFH1-E2 in unprocessed E2-p7 form (Figs 1C and S1). These results validated that SPCS1 facilitates HCV E2-p7 processing in an ectopic protein expression system.

### SPCS1 loss impairs HCV E2-p7 processing during viral replication

**Fig 2:**
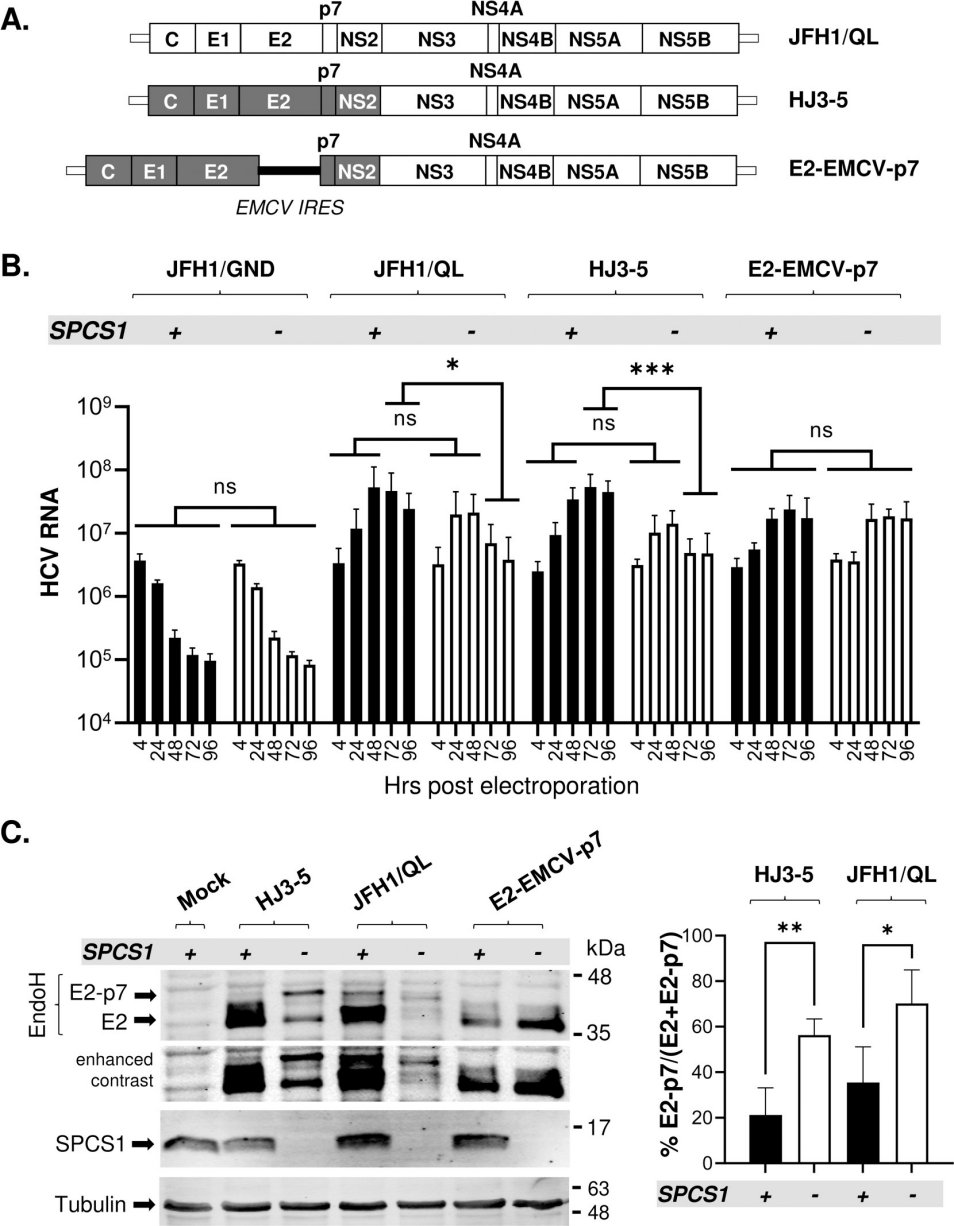
The effect of SPCS1 loss on HCV RNA replication and E2-p7 processing in infectious HCV replicating cells. **(A)** Organization of gt2a JFH1-QL, the gt1a/gt2a HCV chimera HJ3-5 (grey shaded Core to NS2 region from gt1a H77 within the gt2a JFH1 background), and E2-EMCV-p7 encoding the EMCV IRES at the junction site of E2 and p7 in HJ3-5. **(B)** The results of the quantitative TaqMan RT-PCR assays to determine the HCV RNA levels in SPCS1(-) and SPCS1(+) Huh 7.5 cells flowing electroporation of HCV RNAs. The HCV RNA copy numbers at different time points. Results from three biological replicates are shown. **(C)** On the left is the representative western blot analysis of E2 and E2-p7 detected from cell lysates collected at day 2 post-electroporation of the indicated HCV RNAs to SPCS1(-) and SPCS1(+) Huh 7.5. The arrows indicate the locations of E2 and E2-p7. Mean percentages of E2-p7 precursors and the standard deviations from three to five experiments are shown on the right. Statistical analyses were performed by using GraphPad Prism, version 9, software. Asterisks indicate statistically significant differences between two paired values: ***, P < 0.0005; **, P < 0.005; *, P < 0.05. The differences with a P-value of >0.05 were considered not significant (ns).

We assessed whether SPCS1 also regulates E2-p7 processing during HCV replication using three infectious clones of HCV (Fig 2A), including JFH1 with Q221L compensatory mutation in NS3 (JFH1/QL) [42,43], gt1a-gt2a chimera HJ3-5 [44,45], which encodes H77 Core to NS2 region in the JFH1 background, and HJ3-5 derivative E2-EMCV-p7, which encodes internal ribosomal entry site (IRES) from encephalomyocarditis virus (EMCV) between E2 and p7 allowing SPC-independent separation of these two proteins [18].

We first determined the effect of SPCS1 loss in HCV RNA amplification by comparing the relative HCV RNA levels following electroporation of these RNAs into SPCS1(+) or SPCS1(-) cells. HCV RNA levels from replication-defective JFH1/GND (due to NS5B polymerase active site mutation) decreased similarly over time regardless of SPCS1 presence, indicating that SPCS1 loss did not affect HCV RNA stability (Fig 2B). SPCS1 also did not affect replication of infectious HCV RNAs up to 48 hr post-electroporation, consistent with previous reports [35,36]. At 72 to 96 hr time points, we detected near 1 log reduction in JFH1/QL or HJ3-5 RNA levels from SPCS1(-) cells compared to those from SPCS1(+) cells. On the other hand, similar levels of E2-EMCV-p7 RNA were detected at different time points regardless of SPCS1 loss. Since SPCS1 has no impact on subgenomic JFH1 RNA replication (S3 Fig), SPCS1-dependent differential HCV RNA accumulation phenotypes of HJ3-5 and JFH1/QL at later time points are likely due to different effects of SPCS1 loss on infectious virus assembly from these HCV variants (see below).

Anti-E2 western blot of cell lysates collected at day 2 post-electroporation of HJ3-5 or JFH1/QL RNA showed that SPCS1 loss impaired the processing of E2-p7 during viral replication, consistent with the results from the ectopic protein expression system described in Fig 1 (Fig 2C). Interestingly, the efficiency of E2-p7 processing during viral replication was relatively higher than that during ectopic protein expression in SPCS1(+) cells (compare results in Figs 1 and 2C). The presence of p7 downstream sequence in the full-length ORF likely caused this phenotype, as described in the previous report [46]. As expected, only the E2, not the E2-p7 precursor, was detected from E2-EMCV-p7 regardless of SPCS1 loss (Fig 2C). These results suggest that SPCS1 facilitates E2-p7 processing during HCV replication.

### SPCS1 plays a critical role in HCV assembly by facilitating E2-p7 precursor processing during HCV replication

**Fig 3:**
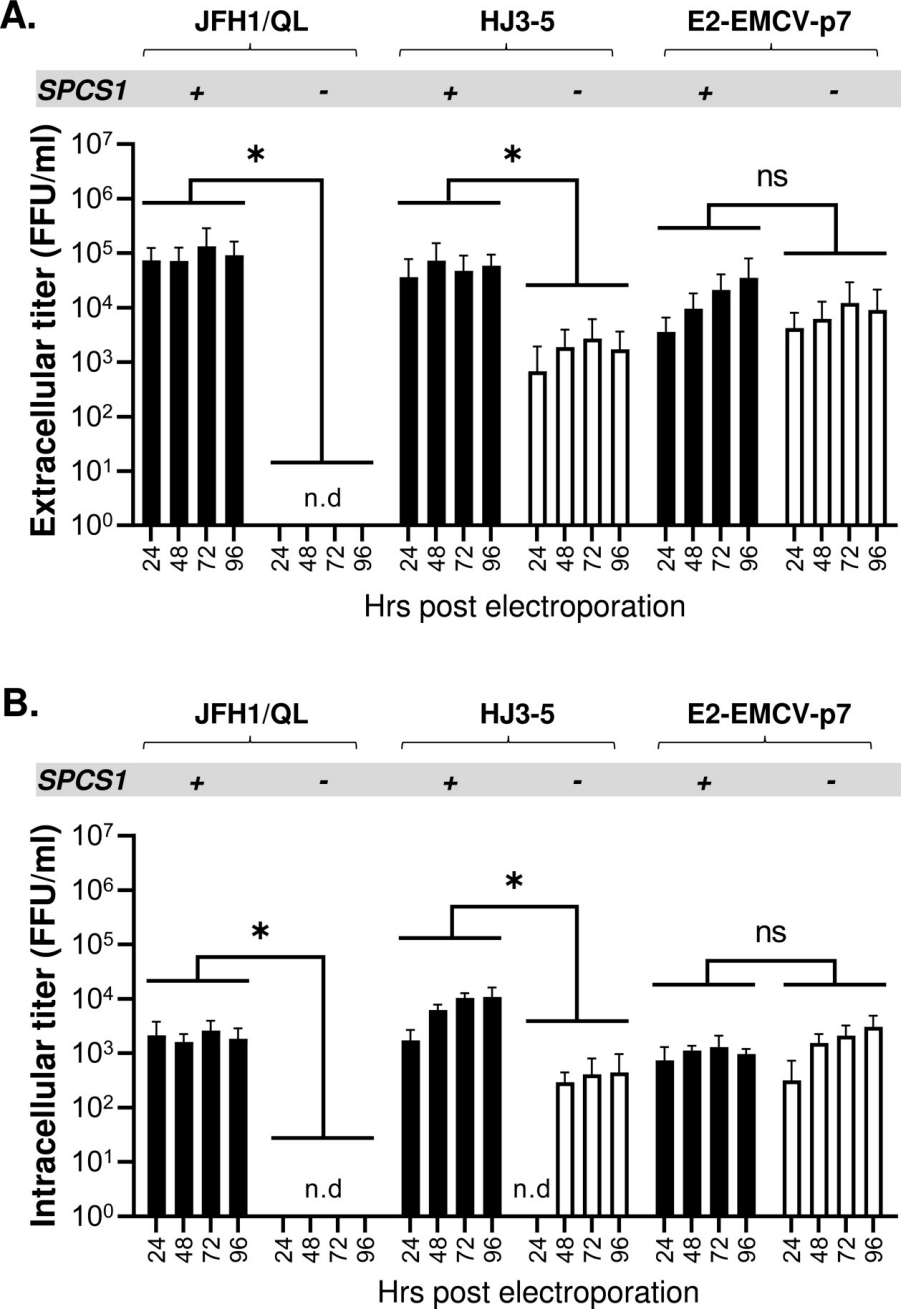
The effects of SPCS1 loss on infectious HCV production. Extracellular **(A)** and intracellular **(B)** virus titers were determined at the indicated time points following electroporation of HCV RNAs, including JFH1/QL, HJ3-5, and E2-EMCV-p7, into the SPCS1(-) and SPCS1(+) Huh 7.5. Mean titers ± standard deviations from three different experiments are shown. The ‘n.d’. denotes not detected. Asterisks indicate statistically significant differences between two paired values: *, P < 0.05. The differences with a P-value of >0.05 were considered not significant (ns).

As described above, the only known role of SPCS1 in HCV assembly has been to enhance the interaction of E2 and NS2, since siRNA knock-down of SPCS1 reduced this interaction [36]. Interestingly, our previous study showed that impairing the E2-p7 processing also inhibited E2 and NS2 interaction, and HCV assembly [18]. Since our results showed that loss of SPCS1 inhibited E2-p7 processing (Figs 1 and 2), we asked whether SPCS1’s role in HCV assembly is contingent on its role in SPC-mediated E2-p7 cleavage. To assess this question, we compared the extracellular and intracellular virus titers derived from HJ3-5 and JFH1/QL, whose assembly requires SPC-mediated E2-p7 processing, and E2-EMCV-p7, whose assembly does not require this step [18]. Consistent with the previous report [33], loss of SPCS1 prevented JFH1/QL virus production since virus infectivity was undetectable either from supernatants or cell lysates up to 96 hr post-electroporation of JFH1/QL RNA into SPCS1(-) cells (Fig 3A and 3B, left side). Loss of SPCS1 also impeded the HJ3-5 virus production resulting undetectable level of intracellular virus production at 24 hr time point, and up to 3 log reductions in extracellular virus titers in SPCS1(-) cells compared to those from SPCS1(+) cells (Fig 3A and 3B, center). On the other hand, SPCS1 showed minimal effect on E2-EMCV-p7 virus production, since intracellular and extracellular E2-EMCV-p7 virus titers derived from SPCS1(+) and SPCS1(-) cells were comparable (Fig 3A and 3B, right side). Minimal effect of SPCS1 loss in infectious E2-EMCV-p7 virus assembly suggests that the major role of SPCS1 in HCV assembly is by facilitating SPC-dependent E2-p7 processing.

**Fig 4:**
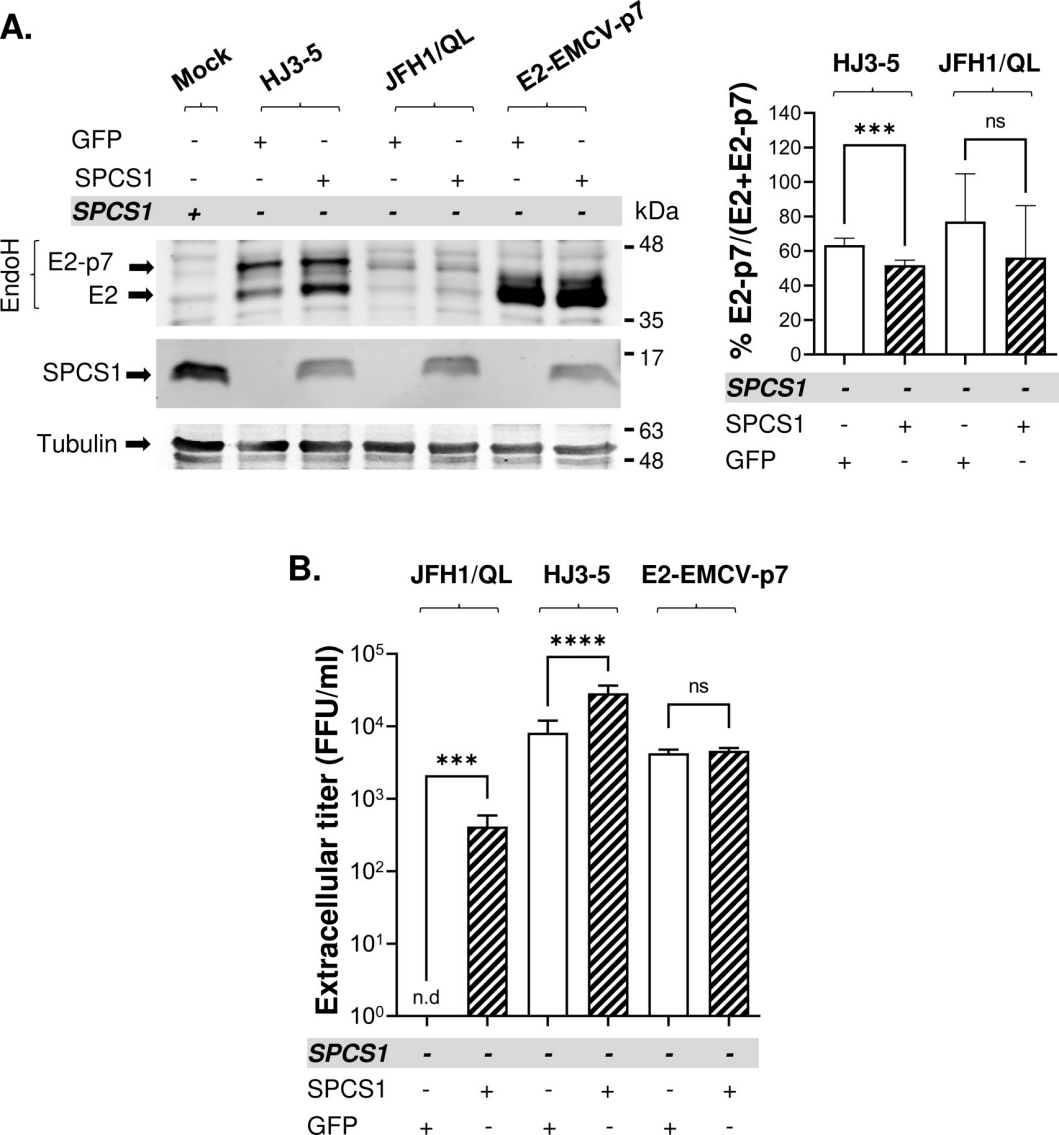
The effect of SPCS1 trans-complementation in SPCS1(-) cells on the E2-p7 processing and virus production. **(A)** On the left is the representative western blot analysis of E2, E2-p7 and SPCS1 detected from cell lysates collected at day 2 post-electroporation of HJ3-5, JFH1/QL or E2-EMCV-p7 RNAs to SPCS1(-) Huh 7.5 cells, which were additionally transfected with the plasmids encoding the SPCS1 or GFP at 6 hr post-electroporation of HCV RNAs. Mock represents non-transfected SPCS1(+) cells. Mean percentages of E2-p7 precursors and the standard deviations from three experiments are shown on the right. **(B)** Extracellular virus titers were determined at day 2 post-electroporation of indicated HCV RNAs, which were additionally transfected with the plasmid*s* encoding the SPCS1 or GFP at 6 hr post*-*electroporation of HCV RNAs. Mean titers ± standard deviations from three different experiments are shown. Asterisks indicate statistically significant differences between two paired values: ****, P < 0.00005; ***, P < 0.0005. The differences with a P-value of >0.05 were considered not significant (ns). The ‘n.d’. denotes not detected.

To verify that SPCS1 indeed mediates the SPC-dependent E2-p7 processing involved in virus assembly, we performed the rescue experiments by transfecting GFP or SPCS1 expressing plasmids at 6 hr post-electroporation of JFH1/QL, HJ3-5, and E2-EMCV-p7 RNA to SPCS1(-) cells. Despite the partial recovery of SPCS1 levels under this experimental condition, H77 or JFH1 E2-p7 processing was enhanced in SPCS1(-) cells replicating HJ3-5 and JFH1/QL, respectively, by SPCS1 transfection, compared to those by GFP transfection (Figs 4A and S2A). SPCS1 transfection also rescued JFH1/QL virus production and significantly enhanced HJ3-5 virus production in SPCS1(-) cells compared to GFP transfection (Fig 4B), suggesting that SPCS1 loss led to impaired the assembly of these viruses in SPCS1(-) cells, consistent with the previous report [36]. Importantly, E2-EMCV-p7 virus titers in SPCS1(-) cells were similar following GFP or SPCS1 transfection, validating that that SPC-independent separation of E2-p7 makes SPCS1 dispensable for E2-EMCV-p7 virus assembly. In aggregate, these results verify that SPCS1 promotes HCV assembly by facilitating E2-p7 processing.

### SPCS1 becomes dispensable for E2-p7 cleavage by relieving the predicted secondary structure of the E2-p7 junction site

**Fig 5:**
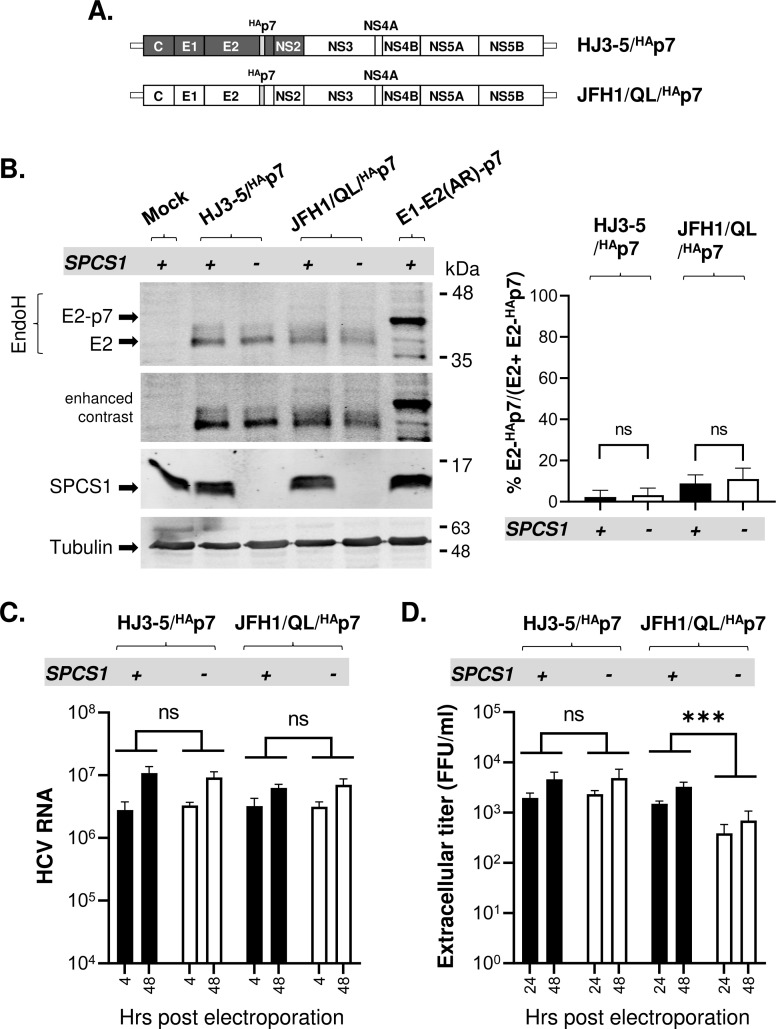
The effect of the HA epitope tag at the N terminus of p7 on SPCS1-dependent E2-p7 processing, HCV RNA replication, and infectious HCV production. **(A)** Organization of the HJ3-5 and JFH1/QL modified to encode the HA epitope tag at the N terminus of p7. **(B)** The western blot detection of E2 and E2-^HA^p7 at day 2 post-electroporation of HJ3-5/ ^HA^p7 and JFH1/QL/^HA^p7 RNAs into SPCS1(+) or SPCS1(-) cells on the left along with the mean percentages of E2-^HA^p7 precursors and the standard deviations from three experiments shown on the right. **(C)** HJ3-5/ ^HA^p7 and JFH1/QL/^HA^p7 RNA copy numbers at 4 and 48 hr post-electroporation of these RNAs into SPCS1(+) or SPCS1(-) cells. **(D)** Extracellular virus titers determined at 24 and 48 hrs post-electroporation of HJ3-5/ ^HA^p7 and JFH1/QL/^HA^p7 RNAs into SPCS1(+) or SPCS1(-) cells. The mean titers are from three biological replicates. Asterisks indicate statistically significant differences between two paired values: ***, P < 0.0005. The differences with a P-value of >0.05 were considered not significant (ns).

Carrere-Kremer *et al*. reported that inserting an HA epitope tag between 3^rd^ and 4^th^ residues of gt1a HCV H strain derived p7 (P3’ and P4’ position of E2-p7 cleavage site) enhances E2-p7 processing using an ectopic protein expression system [17]. Subsequently, we also showed that adding HA epitope tag to N-terminus of H77 p7 enhances E2-p7 processing during HJ3-5 viral replication [18], as evidenced by lack of detectable E2-^HA^p7 precursor (Fig 5B). In this study, we extend this finding by showing that gt2a JFH1 E2-p7 processing is also facilitated by adding HA-epitope to JFH1 p7 N-terminus, as evidenced by detection of only a background level of E2-^HA^p7 precursor (Fig 5B).

**Fig 6:**
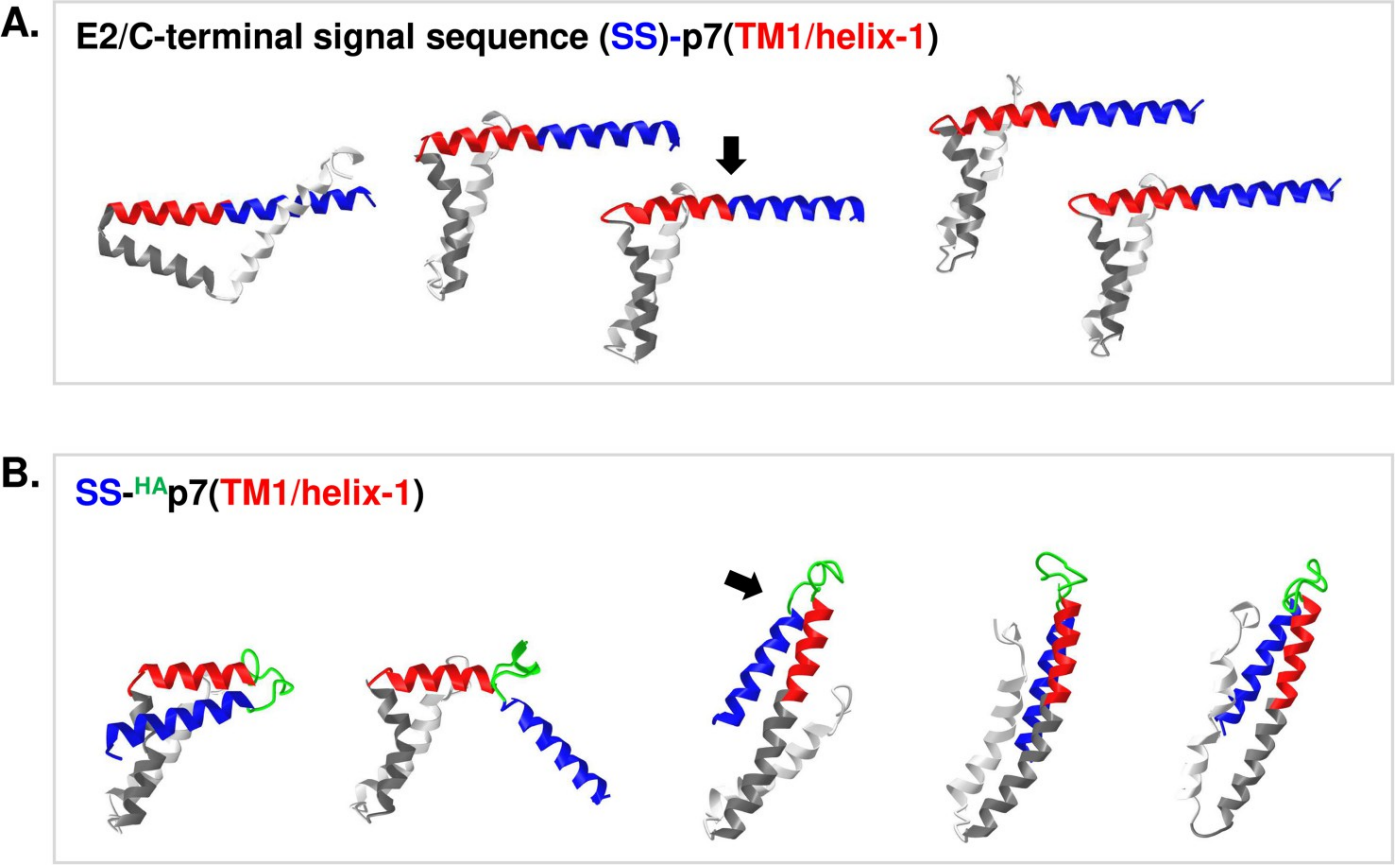
Predicted structures of p7 and ^HA^p7 fused with the leading signal sequence (SS) located at the E2 C-terminus. The five different potential structures of the signal sequence-fused p7 **(A)** without and **(B)** with HA-epitope tag at p7 N-terminus, SS-p7 and SS-^HA^p7, respectively, predicted by the Robetta server [http://robetta.bakerlab.org [47]]. The SS, HA-tag, p7/TM1/helix-1, p7/TM1/helix-2, and the remaining area of the p7 are colored in blue, green, dark grey, and right grey, respectively, by using web-based structure viewer iCn3D (https://www.ncbi.nlm.nih.gov/Structure/icn3d/full.html). The black arrow points to the SPC cleavage site.

**Fig 7:**
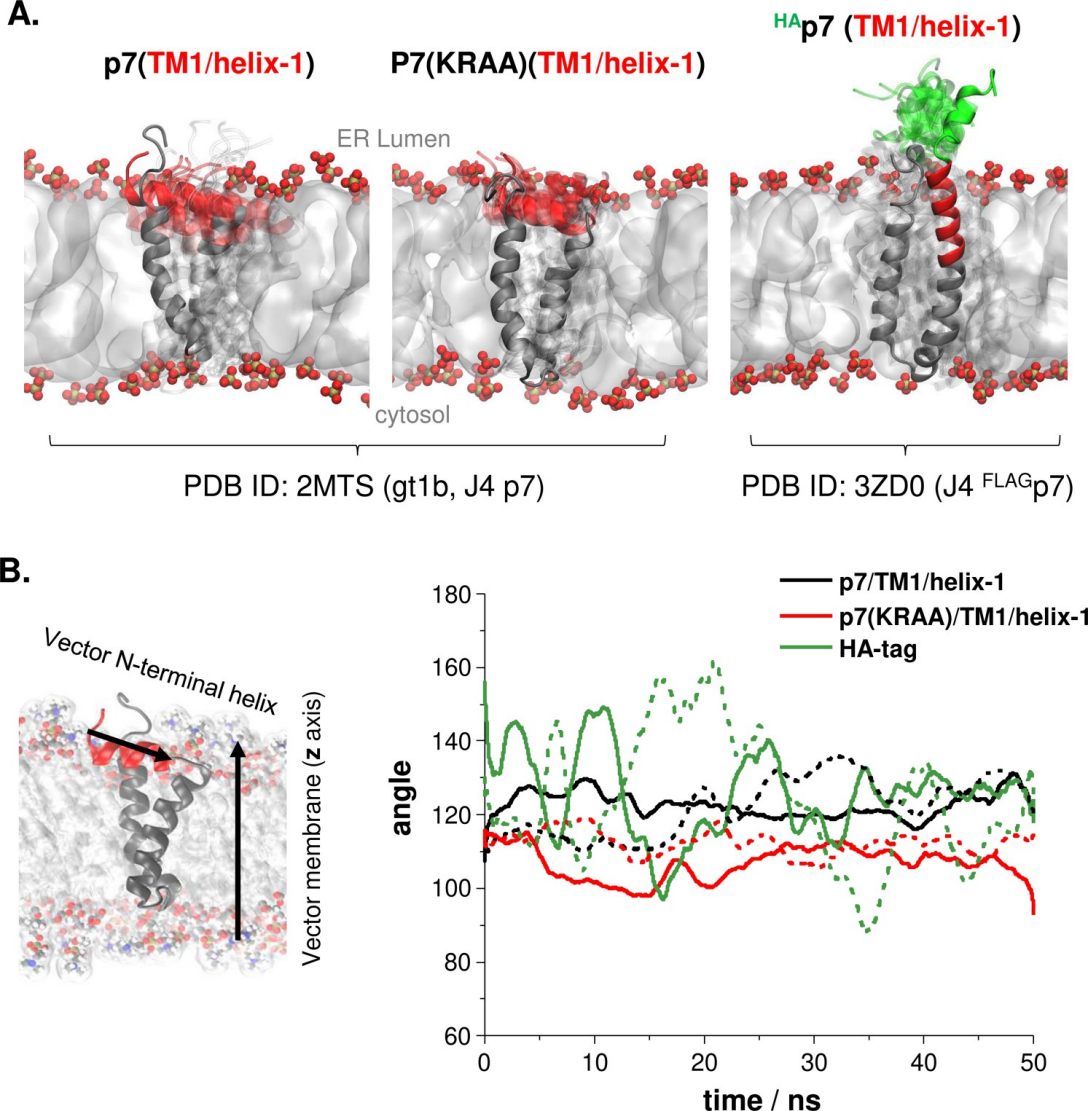
Molecular dynamics simulations of p7 and ^HA^p7 in the membrane environment. **(A)** Snapshots represent the position of the p7/TM1/helix-1 structure along the simulation time (50 ns) for p7 and p7(KRAA) structures based on gt1b J4 p7 (PDB ID: 2MTS) and ^HA^p7 structures based on J4 ^FLAG^p7 (PDB ID: 3ZD0). The p7/TM1/helix-1 and HA-tag helix are highlighted in red and green, respectively, and the positions of these helices at every 5 ns of the simulation time are depicted in translucent. The remaining region of the p7 protein is represented in grey. The membrane core is represented as a surface in white with the lipid heads represented as balls with phosphorous atoms in gold and oxygen in red. **(B)** Representation of the vector used to follow the angle distribution: the vector of the p7/TM1/helix-1 and the vector of the membrane is shown on the left. The p7 protein is represented in grey, with the p7/TM1/helix-1 highlighted in red. The membrane core is represented as a surface in white with the lipid heads represented as balls with carbon atoms in grey, nitrogen in blue, oxygen in red, and hydrogen in white. The angle distribution between the p7/TM1/helix-1 or HA-tag helix structures and the membrane normal along the simulation time is shown on the right. The different protein structures tested are represented with different colors; wt p7/TM1/Helix-1 in black, p7(KRAA) /TM1/Helix-1 mutant in red, and HA-tag in ^HA^p7 in green. The solid and dashed lines represent two different simulations, and data were smoothed using adjacent averaging with 200 points per window.

Carrere-Kremer *et al*. suggested that HA-tag insertion releases structural constraint of E2-p7 junction site leading to improved cleavage at this site [17]. Fig 6 shows the structures of p7 fused with signal sequence (SS) located at the E2 C-terminus without and with HA-epitope tag at p7 N-terminus, SS-p7 and SS-^HA^p7, respectively, predicted by Robetta server (http://robetta.bakerlab.org) [47]. The predicted SS-p7 structure (represented by 4 out of 5 models) is remarkable for two aspects that are consistent with suboptimal E2-p7 processing. First, the E2-p7 cleavage site is in the middle of the alpha-helix extending from the SS (colored in blue) into the first half of the p7 transmembrane domain 1 (p7/TM1/helix-1, colored in red) (Fig 6A). This type of secondary structure at the SPC cleavage site was shown to inhibit SPC-mediated cleavage [15]. Second, due to the bending of p7/TM1/helix-1 from the following p7/TM1/helix-2 (colored in dark grey), resembling that in p7 structure described by Cook *et al*. [48], the E2-p7 cleavage site would lie within or close to the surface of the membrane (see Fig 7 for the location of the bent helix-1 relative to the membrane). Since the active site of signal peptidase is located at the ER lumen, about 0.4–1.1 nm above the ER membrane surface [15,49,50], the proximity of E2-p7 cleavage site to the membrane surface, by decreasing its chance of encountering the SPC active site, likely have contributed to the delayed cleavage of E2-p7 junction by SPC. On the other hand, the predicted signal sequence (SS)-^HA^p7 structure (represented by 3 out of 5 models) showed the ideal exposure of its cleavage site to the SPC active site, due to the helix breaking nature of HA-tag and extension of p7/TM1/helix-1, similar to that in p7 structure described by Montserret *et al*. [51] and Foster *et al*. [52] (Fig 6B), presenting the N-terminal of HA-tag above the membrane surface into the ER lumen (Fig 7A).

We asked whether the structural constraint surrounding the E2-p7 junction necessitates the SPCS1-specific role for SPC-mediated cleavage of this site. To address this question, we assessed whether the E2-^HA^p7 precursor, lacking the structural constraint of the E2-p7 junction region, still requires SPCS1 for its processing during replication of HJ3-5/^HA^p7 and JFH1/QL/^HA^p7 viruses. In SPCS1(+) cells, both H77- and JFH1-E2-^HA^p7 precursors were efficiently processed (Fig 5B). Importantly, these precursors were also efficiently processed in SPCS1(-) cells, indicating that SPCS1 is dispensable for E2-^HA^p7 processing (Fig 5B). These results suggest that SPCS1 is required for SPC-mediated processing of the E2-p7 junction site due to the structurally unfavorable nature of this site as a signal peptidase substrate.

Next, we determined the impact of efficient, SPCS1-independent E2-^HA^p7 processing on HJ3-5/^HA^p7 and JFH1/QL/^HA^p7 viral RNA replication and assembly. In the case of HJ3-5/^HA^p7, its RNA replication and virus assembly were not affected by SPCS1 loss (Fig 5C and 5D), mimicking the phenotypes displayed by E2-EMCV-p7 (Figs 2B, 3A and 3B). These results suggest that efficient separation of gt1a H77 E2 and p7, regardless of the involvement of SPC-mediated processing, makes SPCS1 dispensable for virus production.

In the case of JFH1/QL/^HA^p7, however, while SPCS1 loss also had no impact on viral RNA replication (Fig 5C), its viral assembly was significantly lower in SPCS1(-) cells compared to that in SPCS1(+) cells (Fig 5D). In other words, efficient, SPCS1-independent E2-p7 processing could only partially restore the SPCS1 loss-mediated virus production defect in JFH1/QL (Figs 3B and 5B). These results suggest that SPCS1 may have an additional, JFH1-specific role in virus assembly (see below).

### The p7 mutation that inhibited E2-p7 processing induced a narrower p7/helix-1 bending angle against the z-axis of the membrane

We performed molecular dynamics (MD) simulation of p7 in a model membrane to characterize the behavior of p7 without or with HA-tag in its natural environment. As for wild type (wt) p7, we used a p7 structure [Protein Data Bank (PDB) ID: 2MTS] described by Cook *et al*. [48], which represents the helix-1-bended form of p7, resembling the p7 structure predictions by the Robetta server shown in Fig 6A. To understand the p7 conformation dynamics that restrict the E2-p7 processing, we also determined the effect of p7(KRAA) mutations, which impaired the E2-p7 processing [18], by introducing these mutations to the 2MTS structure before MD simulations. We focused on identifying the difference in p7/TM1/helix-1 movement between wt p7 and p7(KRAA) mutant, since this region serves as a major determinant for delayed E2-p7 processing, as discussed above. The p7(KRAA) mutant, among different E2-p7 processing impairing mutants, were chosen in this analysis, since p7 residues 33 and 35 mutated in this mutant, are located at the cytoplasmic loop area between the two TM domains of p7, away from the p7/TM1/helix-1. To generate the ^HA^p7 structure, we replaced the FLAG-tag sequence with the HA-tag sequence in the ^FLAG^p7 structure (PDB ID: 3ZD0, Foster *et al*. [52]), which represents the helix-1-upright form of p7 resembling structure predictions from the Robetta server shown in Fig 6B. Interestingly, under this condition, part of the HA-tag folds into an α-helix (Fig 7A), rather than an unfolded coil state, as predicted by the Robetta server (Fig 6B).

We performed MD simulations of these p7 derivatives embedded in a model membrane composed of POPC (1-palmitoyl-2-oleoyl-sn-glycero-3-phosphocholine) (see Materials and Methods for detail). To quantitatively compare the position of p7/TM1/helix-1 of wt p7 and p7(KRAA) mutant, we assessed the position of this region relative to the membrane normal (z-axis) by determining the distribution of the angle between the vector from the C-alpha atom of the first residue to the last residue of the p7/TM1/helix-1 and the membrane normal vector (Fig 7B, left panel), along the 50 ns simulation time. The 90° angle would mean that p7/TM1/helix-1 is fully folded into the membrane (perpendicular to the membrane normal) and the 180° angle would indicate that it is fully extended into the ER lumen. The wt p7/TM1/helix-1 (colored in red) showed relatively limited movement within the membrane environment, maintaining the position nearly perpendicular to the membrane normal (*approx*. 110° to 130°) (Fig 7A, left panel, and Fig 7B, right panel). Interestingly, as shown in snapshots along the 50 ns simulation time, p7(KRAA)/TM1/helix-1 (colored in red) showed an even more limited movement within the membrane environment (Fig 7A, middle panel), when compared to wt p7/ TM1/helix-1 (Fig 7A, left panel). Furthermore, p7(KRAA)/TM1/helix-1 maintained the angle even closer to 90° (100° to 120°), indicating that TM1/helix-1 in p7(KRAA) mutant is more embedded in the membrane than that in wt (Fig 7B, right panel). These results suggest that a relatively stable association of the p7/TM1/helix-1 with the membrane is one of the mechanisms for delayed E2-p7 processing by inhibiting the effective presentation of the E2-p7 junction to the SPC catalytic site located in the ER lumen well above the membrane. In the case of the p7(KRAA) mutant, increased membrane-embedded nature of the p7/TM1/helix-1 likely further inhibited the E2-p7 junction exposure to the ER lumen, leading to negligible processing of this site by the SPC.

Meanwhile, the HA-tag (colored in green) shows a more variable angle distribution relative to the membrane along the simulation time, ranging from *approx*. 100° to 160°. This indicates that the HA-tag fused to p7-N-terminus fluctuates more during equivalent simulation time, ranging from conformations where it is almost perpendicular to the membrane to conformations where it is almost fully extended to the ER lumen (Fig 7A, right panel, and Fig 7B, right panel). The freedom of movement of the HA-tag in the ER lumen likely facilitated the SPC-mediated cleavage between E2 and HA-tag during HJ3-5/^HA^p7 and JFH1/QL/^HA^p7 virus replication (Fig 5B).

Both p7 structures used in the above MD simulations were derived by using HCV J4 strain (gt1b) p7 protein with or without FLAG epitope tag (PDB ID: 2MTS and 3ZD0, respectively). To better understand the effect of different p7 sequences on the rigidity of the p7/TM1/helix-1, as well as the movement of the HA epitope tag at the p7 N-terminus within the membrane environment, we replaced the J4 p7 sequence in the above structures with those of H77 or JFH1 and repeated the MD simulations. Despite the substantial sequence difference between J4- and H77- or JFH1-p7 (13 and 26 different amino acids, respectively), the p7/TM1/helix-1 of H77 and JFH1 displayed the limited movement and maintained a position nearly perpendicular to the membrane normal (S5 Fig), similar to that of J4-p7 (Fig 7). Interestingly, H77 p7/TM1/helix-1 (solid and dashed lines colored in black) was more embedded inside the membrane (*approx*. 100° on average), compared to JFH1 p7/TM1/Helix-1 (solid and dashed lines colored in blue, *approx*. 120° on average) (S5C Fig, right panel). These data imply that the processing of H77 E2-p7 may be more inefficient than that of JFH1, consistent with the relatively higher average level of H77 E2 in E2-p7 precursor form than that of JFH1 in the absence of SPCS1 (Fig 1A and 1B).

Similar to the case of J4 ^HA^p7 MD simulations above, HA-epitope tag fused to H77 or JFH1 p7 N-terminus fluctuated more during the simulated time, ranging from conformations almost perpendicular to the membrane to those almost fully extended into the ER lumen (angles ranging from *approx*. 90° to 170°) (S5 Fig). The behaviors of HA epitope tag in H77 ^HA^p7 (lines colored in green) and JFH1 ^HA^p7 (lines colored in brown) were very random for each simulation repetition (solid lines versus dashed lines), making it difficult to distinguish whether either of their HA tags tends to adopt more time exposed to the ER lumen. These data are consistent with the equally efficient, SPCS1-independent, SPC-mediated cleavage of H77- and JFH1-E2-^HA^p7 shown in Fig 5B.

### SPCS1 preferentially interacts with the E2-p7 precursor

**Fig 8:**
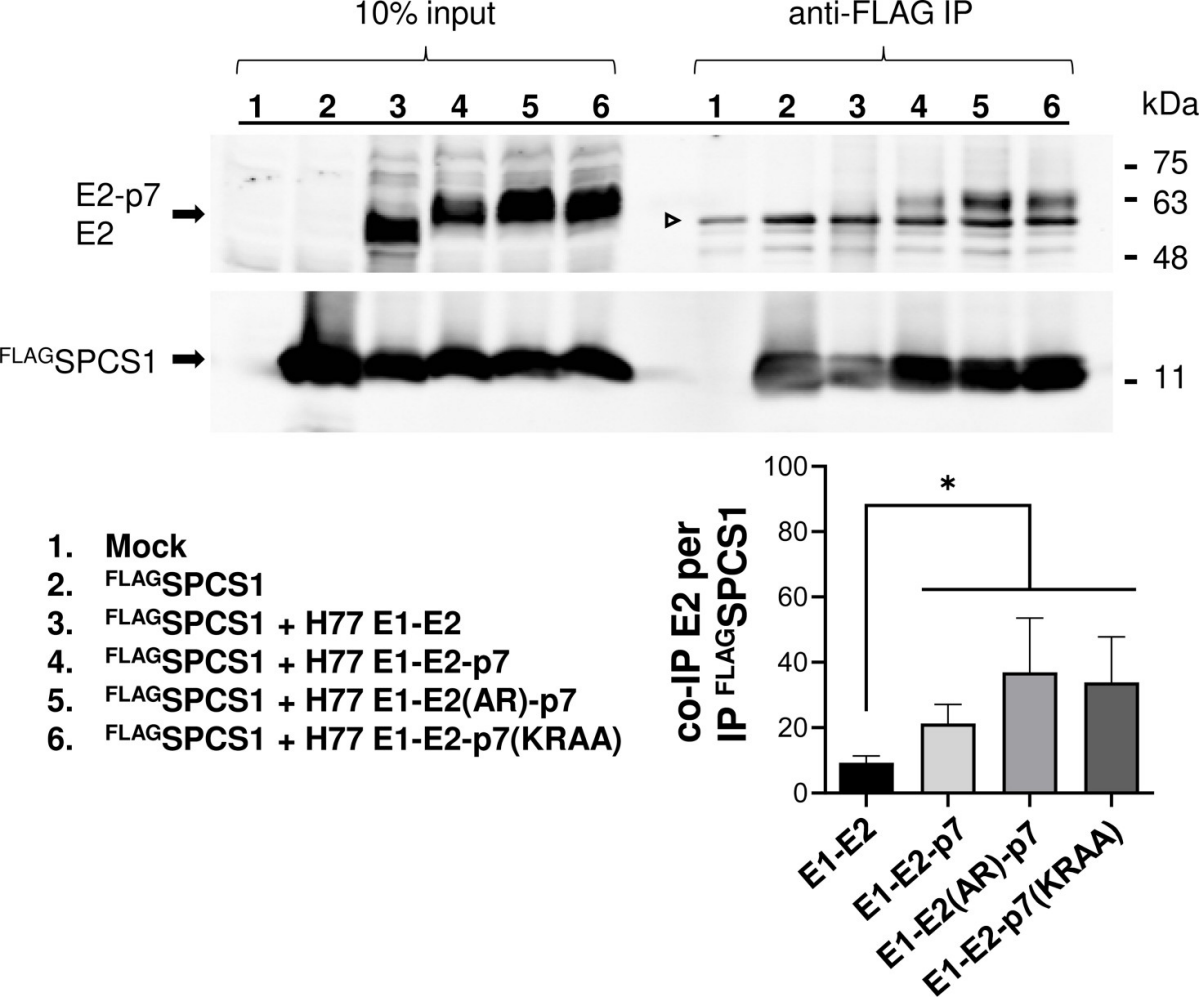
Interaction of SPCS1 with HCV E2 or E2-p7 precursor. The interaction between ^FLAG^SPCS1 and E2 derivatives was determined by anti-FLAG antibody-mediated co-immunoprecipitation (co-IP) assay following co-transfection of ^FLAG^SPCS1 without or with H77 E1-E2, E1-E2-p7, E1-E2(AR)-p7, and E1-E2-p7(KRAA) to 293T cells. The E2 co-IP efficiency was calculated by dividing the relative ratio of co-immunoprecipitated E2 derivatives normalized by the input E2 derivatives by the relative ratio of immunoprecipitated ^FLAG^SPCS1 normalized by the input ^FLAG^SPCS1. The arrowhead indicates the location of the antibody heavy chain. Mean percentages of E2 co-IP efficiency and the standard deviations from four experiments are shown at the bottom right. Asterisks indicate statistically significant differences between two paired values: *, P < 0.05.

Although SPCS1 was shown to interact with HCV E2 [36], it is unknown whether it could interact with the E2-p7 precursor. If the role of SPCS1 is to relieve the structural constraint present at the E2-p7 junction site, it is reasonable to expect the SPCS1 to interact with the E2-p7 precursor. To determine the interaction between SPCS1 and E2-p7 precursor, we co-transfected FLAG-tagged SPCS1 (^FLAG^SPCS1) and E2 derivatives, into HEK293T cells, and performed co-immunoprecipitation (co-IP) experiments using anti-FLAG antibody (Fig 8). This strategy is adopted since the anti-SPCS1 antibody lacked the capacity to immunoprecipitate endogenous SPCS1. We performed the experiments using E2-p7 derivatives that showed partial and complete inhibition of E2-p7 processing including E1-E2-p7, and E1-E2(AR)-p7 and E1-E2-p7(KRAA), respectively. The E1-E2 was used as a negative control lacking the E2-p7 precursor. As shown in Fig 8, SPCS1 preferentially interacts with E2-p7 precursor rather than processed E2 since the average pull-down efficiency of E2 derivatives by the SPCS1 inversely correlates with E2-p7 processing efficiency. These results are consistent with a recent finding by Yim *et al*., who also demonstrated the SPCS1-mediated pull-down of uncleaved, signal-anchored protein [34].

### SPCS1 has a genotype-specific role in JFH1 assembly by regulating JFH1 NS2 stability

**Fig 9:**
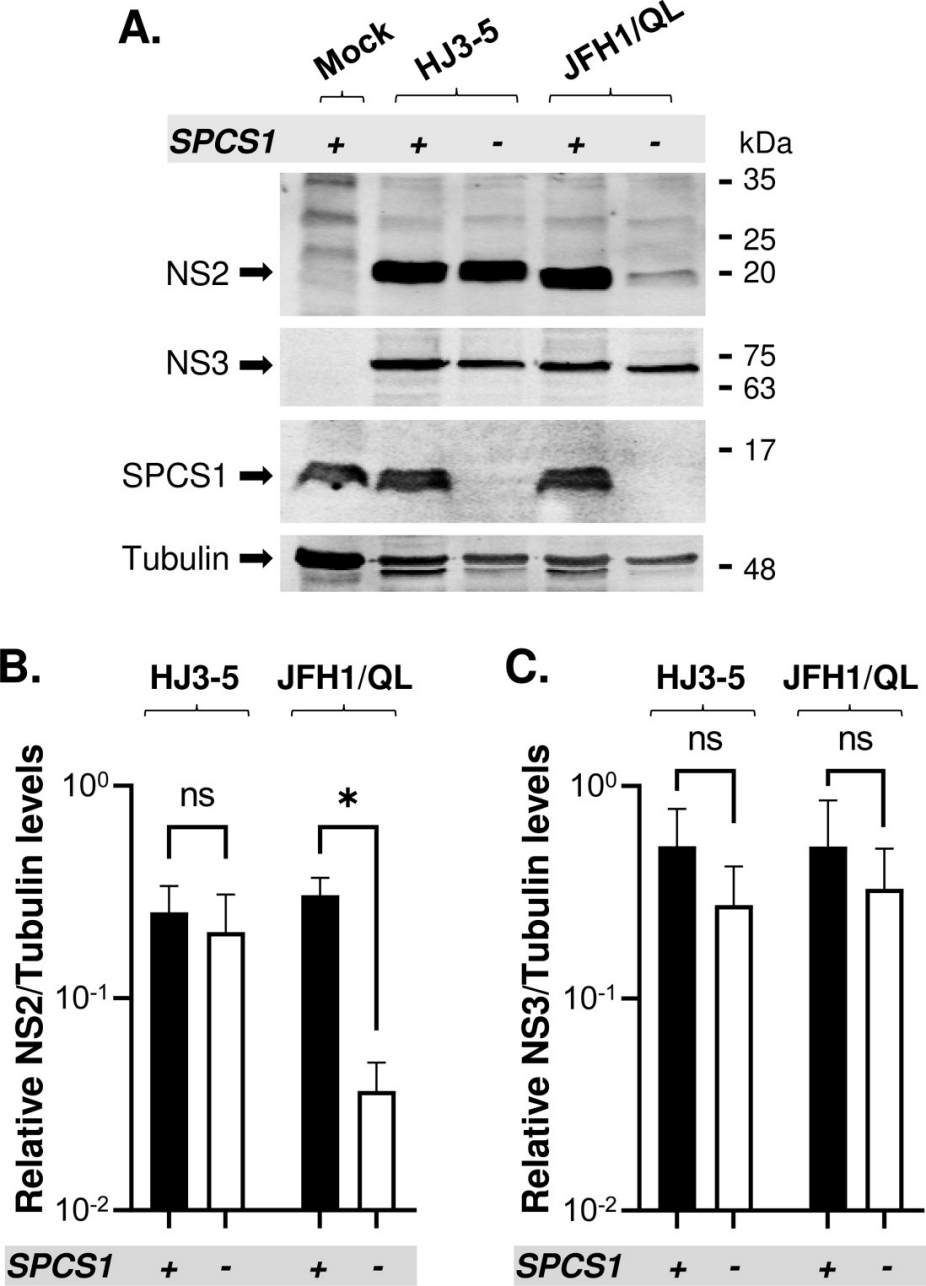
Effect of SPCS1 loss on NS2 and NS3 levels during replication of HJ3-5 and JFH1/QL. **(A)** The western blot detection of NS2 and NS3 at day 2 post-electroporation of HJ3-5 and JFH1/QL RNAs into SPCS1(+) or SPCS1(-) cells. The relative levels of NS2 **(B)** and NS3 **(C),** normalized by those of Tubulin in experiments above. Asterisks indicate statistically significant differences between two paired values: *, P < 0.05. The differences with a P-value of >0.05 were considered not significant (ns).

As shown in Fig 3, SPCS1 loss prevented JFH1/QL virus production while partially permitting HJ3-5 virus production. SPCS1-independent E2-p7 separation, either by introducing EMCV IRES between E2 and p7 (Fig 3) or adding HA tag to the p7 N-terminus (Fig 5), either completely or partially restored virus production from HJ3-5 or JFH1/QL, respectively. These results suggest that SPCS1 has the genotype-specific function(s) on JFH1 assembly, in addition to its common, genotype-independent role in E2-p7 processing. We determined that SPCS1 has no impact on either H77- or JFH1-p7-NS2 processing as evidenced by lack of accumulation of uncleaved p7-NS2 at the predicted size of this precursor (Fig 9A), consistent with previous finding [36]. Interestingly, SPCS1 loss specifically reduced the steady-state level of JFH1 NS2, but not that of H77 NS2, while having no impact on either JFH1 NS3 or H77 NS3 levels during replication of JFH1/QL and HJ3-5, respectively (Fig 9), despite that SPCS1 could interact with both H77- and JFH1-NS2 (S4 Fig). Since NS2-mediated HCV assembly complex formation is critical for infectious virus production [53–56], SPCS1-mediated regulation of JFH1 NS2 stability could account for the JFH1/QL virus assembly-specific role of SPCS1.

## Discussion

SPCS1 is one of the regulatory subunits of the eukaryotic signal peptidase complex (SPC) [24,27,28]. Flaviviridae family members, including HCV, require SPCS1 for efficient propagation [33,35,36]. SPCS1-dependency in flavivirus production is attributed to its role in regulating SPC-mediated C-prM processing [33], in addition to its interaction with NS2B [37]. However, until now, the only known function of SPCS1 in HCV assembly has been its interaction with NS2 and E2 enhancing their interaction [36]. In this study, we determined that 1) HCV assembly depends on SPCS1 to specifically promote SPC-mediated E2-p7 processing and 2) SPCS1 dependence of HCV assembly could be completely or significantly bypassed (depending on HCV genotypes) either by separating E2 and p7 in an SPC-independent manner or by adding an epitope tag, enhancing SPC-mediated processing of this junction site. These results indicate that one of the major functions of SPCS1 in HCV assembly is to regulate the efficiency of E2-p7 processing by SPC. Accordingly, this study establishes the common role of SPCS1 in Flaviviridae family virus propagation as to exquisitely regulate the SPC-mediated processing of specific, suboptimal target sites, E2-p7 junction in HCV, just as previously described C-prM junction in flavivirus [33], whose processing efficiency plays a key role in infectious virus assembly (see recent review by Alzahrani *et al*. [19] for detail on how processing efficiency at these sites regulate virus assembly).

Despite being translated continuously from a single open reading frame and processed by the same SPC, unlike preceding co-translational processing of Core-E1 and E1-E2 junctions, E2-p7 processing is delayed, post-translational event [5,6,17–20]. Likely, the transmembrane (TM) domain(s) of the post-translational SPC substrates such as HCV E2-p7 will be released from the translocon to the ER membrane first, allowing continuous translation of the remaining coding region. Then, SPCS1 will preferentially recognize and interact with these uncleaved substrate precursors, as shown in Fig 8 and described previously by Yim *et al*. [34]. Next, SPCS1 interaction with these substrates may facilitate the signal sequence cleavage site presentation to the catalytic site of SPC located in the ER lumen enhancing the processing of these substrates by the SPC. The post-translational processing by the signal peptidase could occur *in trans* as demonstrated by Jackson and Blobel [57], supporting this potential sequence of events.

This delayed processing of E2-p7 must be regulated in a perfectly balanced manner for optimal HCV propagation since any alteration of E2-p7 processing, either promoting or inhibiting it, led to impaired HCV assembly [18,22,23]. Consistent with the narrow range of ideal E2-p7 processing efficiency, the SPCS1’s role in SPC-mediated E2-p7 processing was not drastic. Complete SPCS1 loss reduced, not prevented, SPC-mediated E2-p7 processing (Figs 1 and 2). On the other hand, even in the presence of SPCS1, E2-p7 processing was not complete (Figs 1 and 2). Importantly, SPCS1 became dispensable for E2-p7 processing by the insertion of HA epitope tag to p7-N-terminus, which facilitated SPC-mediated processing at this site by relieving structural constraints at the E2-p7 junction (Figs 5, 6 and 7). These results indicate that HCV depends on the host factor SPCS1 to orchestrate the delicate balance in SPC-mediated processing of E2-p7 junction, of which suboptimal SPC substrate status is imposed by structural determinants intrinsic to the p7 N-terminal sequence [17,23] (Fig 7).

Our results suggest that SPCS1 has HCV genotype-independent role in virus assembly by facilitating E2-p7 processing. However, gt2a JFH1/QL assembly additionally depended on SPCS1 due to the genotype-specific role of SPCS1 on JFH1 NS2 stability (Fig 9). Interestingly, our previous study showed that the stability of H77 NS2, but not that of JFH1 NS2, is regulated by a proteasome degradation system [45]. These results suggest that HCV utilizes different mechanisms to regulate the stability of multifunctional protein NS2, involved in both viral RNA replication and assembly [53–56,58], in a genotype-dependent manner.

**Fig 10:**
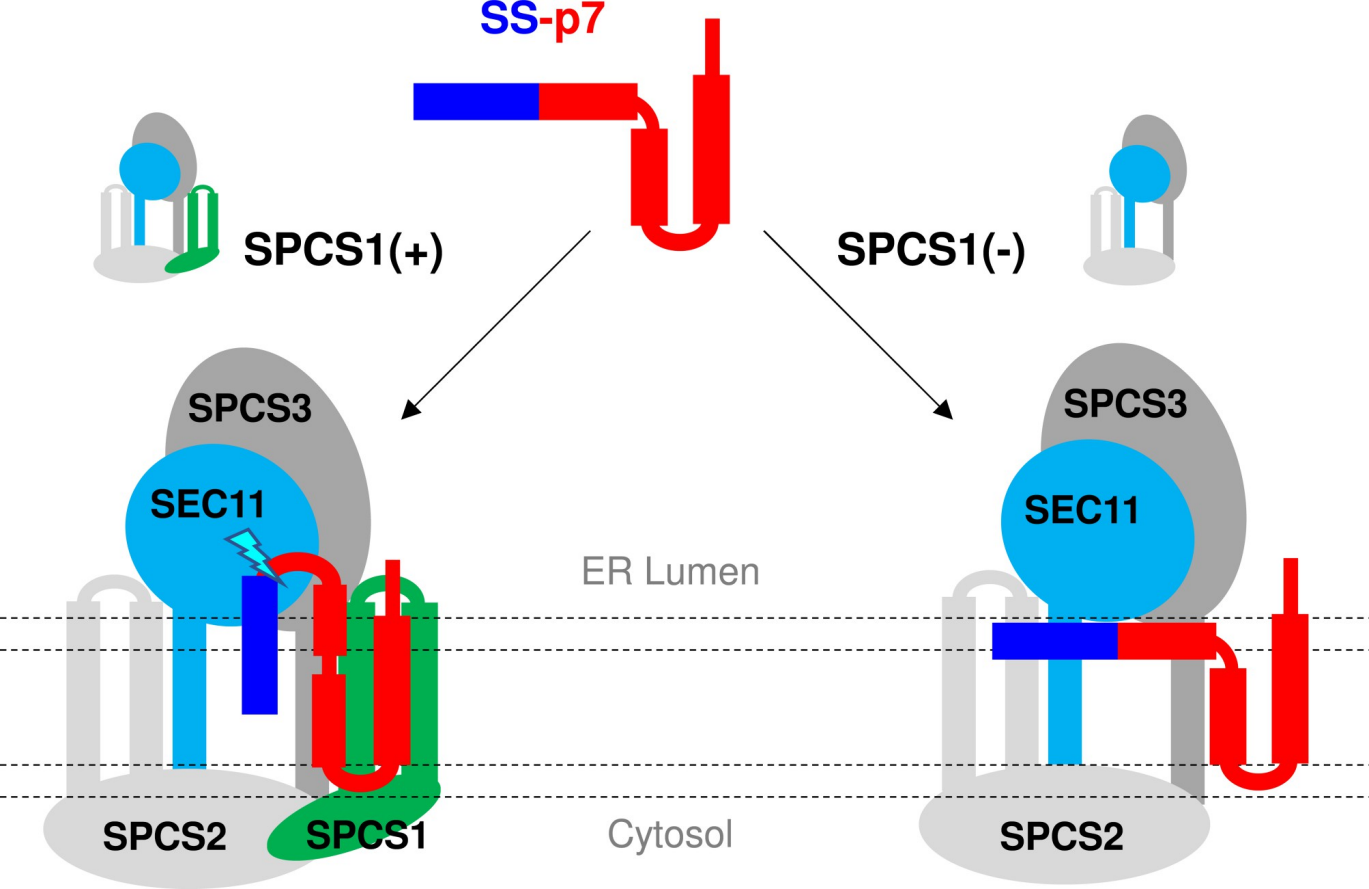
Model for the potential role of SPCS1 in regulating HCV E2-p7 processing. We propose that SPCS1 positively regulates the signal peptidase complex (SPC) processing of the HCV E2-p7 junction by facilitating the presentation of the cleavage site, an unfavorable substrate for the SPC due to structural constraints and membrane localization, to the SPC catalytic site. Cartoon representations for the signal sequence (SS)-p7, SPC, and the placement SS-p7 on the SPC are for model demonstration only and may not represent actual structures or interactions.

The study by Oestringer *et al*. suggested that p7 from different genotypes could form two alternative conformations resembling those shown in Figs 6 and 7, with p7/TM1/helix-1 in either bent or extended states relative to the membrane z-axis [48,51,52,59,60]. Our MD simulation shows that p7/TM1/helix-1 in a bent form associates with the proximal leaflet of the membrane nearly perpendicular to the membrane normal, maintaining a relatively stable position along the simulation time (Fig 7B). According to the structure prediction by the Robetta server, the prevailing state of the p7/TM1/helix-1 in an SS-p7 precursor form is a bent form as part of a continuous α-helix formed by SS and the p7/TM1/helix-1 (Fig 6A). If this predicted structure is true, the E2-p7 cleavage site will be embedded in the membrane, making it an unfavorable substrate for the SPC (Fig 7). In addition, α-helical structure in the c-region of the signal sequence will further impede the SPC-mediated E2-p7 processing. We propose that SPCS1 may increase the p7/TM1/helix-1 angle relative to the membrane normal to facilitate the E2-p7 junction substrate presentation to the SPC catalytic site, leading to enhanced E2-p7 processing (Fig 10). Since helix to coil transition might be possible upon hydration of the linker helix [61], even if E2-p7 cleavage junction forms the α-helical structure as predicted, exposure of this junction to the ER-lumen by the SPCS1 could lead to the hydration and helix to coil transition of this region making this junction a better substrate for SPC cleavage. This model is supported by our finding that SPCS1 becomes dispensable for SPC-mediated cleavage of E2-^HA^p7 junction, which is predicted to be exposed to the ER lumen due to an HA-tag providing helix breaking coiled region to the cleavage site (Figs 5B, 6B and 7).

Our MD simulation data showed that p7(KRAA) mutations that impaired the E2-p7 processing reduced the p7/helix-1 angle relative to membrane normal making this region more membrane-embedded. Interestingly, Scull *et al*. showed that replacing p7-TM1/helix-1 residues with the bulky, hydrophobic Tryptophan impaired the E2-p7 processing and virus production [23]. These Tryptophan substitutions likely increased the hydrophobicity of p7/TM1/helix-1, strengthening the membrane association of these mutants. Notably, both KRAA mutations and Tryptophan substitution mutations in p7 impaired the E2-p7 processing in the SPCS1(+) cells. We speculate that the p7/TM1/helix-1’s membrane-association-angle change caused by the KRAA mutations, and the likely increase in membrane association affinity of the p7/TM1/helix-1 caused by the Tryptophan substitution, may have induced the sufficient barrier against the ER luminal exposure of E2-p7 junction, which is too high to overcome even by the action of the SPCS1.

Similar to HCV E2-p7 processing, the SPC-mediated cleavage of flavivirus C-prM junction is a delayed, post-translational event requiring prior cleavage of C protein by NS2B/NS3 protease after the two basic residues preceding the transmembrane domain (*anchor C*), which serves as a signal sequence [38–41, 62, 63]. SPCS1 loss inhibited the *anchor C*-prM cleavage, and SPCS1 dependence of this cleavage could be bypassed by replacing the *anchor C* sequence with a signal sequence of the major histocompatibility complex (MHC) antigen [33]. These results suggest that SPCS1 is required for SPC-mediated C-prM cleavage to compensate for the defect in the *anchor C* sequence. The signal peptide is composed of a positively charged n-region followed by a hydrophobic h-region and a polar c-region containing the SPC cleavage scissile bond [64]. Unlike the signal sequence for HCV E2-p7, which is effective on its own [17], the flavivirus *anchor C* signal sequence is defective due to the lack of polar residue at the c-region [38]. Due to this hydrophobic nature of the c-region, the *anchor C* sequence, including the c-region, is predicted to form a transmembrane domain with the cleavage site buried in the membrane when preceding C protein is covalently attached [38]. The membrane association of the c-region will likely prevent the ready access of this cleavage site to the SPC active site located well above the membrane. It is remarkable that the common feature of HCV E2-p7 junction and flavivirus C-prM junction, which requires SPCS1 to promote SPC-mediated cleavage, is the membrane-associated nature of these cleavage sites which will inhibit their ready access to SPC active site. These results suggest that mechanism of action of SPCS1 is to facilitate the hydrophobic substrate processing by the SPC.

In conclusion, our study revealed that SPCS1 is required to facilitate HCV E2-p7 processing to promote HCV assembly. Our study also suggests that SPCS1 is required to increase the membrane-associated substrate recognition by the SPC active site. The SPCS1-dependent, suboptimal processing of HCV E2-p7 and flavivirus C-prM by the SPC is critical for the common purpose of nucleocapsid envelopment [19]. Our study suggests that these two viruses belonging to two different genera in the same Flaviviridae family accomplish these highly regulated processes by exploiting the specialized function of SPCS1 in SPC-mediated processing using a common membrane-associated substrate feature of different precursors, generated by different mechanisms.

## Materials and methods

### Cells

Huh-7 cell line clonal derivatives, including Huh-7.5 [65] and FT3-7 cell lines [66] were used, as well as control SPCS1(+) and SPCS1 knock-out SPCS1(-) CRISPR-Cas9 edited Huh-7.5 cells (Provided generously by Dr. Michael Diamond in Washington University, [33]). These were maintained in Dulbecco’s modified Eagle medium (DMEM) (Invitrogen, Carlsbad, CA) containing 10% fetal bovine serum (Invitrogen, Carlsbad, CA) at 37°C in a 5% CO_2_ environment.

### Plasmids

HJ3-5, JFH1-QL, and HJ3-5/^HA^p7 were described previously [18,67,68]. JFH1/^HA^p7 was generated by inserting the HA-epitope sequence between the E2 and p7 coding region of JFH1/QL. The pFN11A (BIND) Flexi Vector (Promega) was modified by deleting the GAL4 fusion protein-coding region and used to generate plasmids expressing E1-E2, E1-E2-p7, E1-E2(AR)-p7, and NS2 from two different genotypes, H77 and JFH1. In brief, E1-E2, E1-E2-p7, E1-E2(AR)-p7, NS2 sequences were PCR amplified from H77 and JFH1 with the primer sets introducing SgfI and PmeI restriction enzyme sites at their N- and C-terminus, respectively, and then cloned into the modified vector digested with SgfI and PmeI enzymes (Promega, WI, USA). The E2 (AR) mutations were introduced by using the QuikChange II XL site-directed mutagenesis kit (Agilent Technology, Santa Clara, CA). The sequences of regions manipulated within each plasmid were verified by DNA sequencing. The SPCS1 sequence was cloned from cellular RNA isolated from Huh-7 cells and inserted into the modified pFN11A vector described above, without or with Flag- or HA-tags.

### DNA transfection

12-well plates were seeded with SPCS1(-) or SPCS1(+) Huh 7.5 cells in DMEM containing 10% fetal bovine serum (FBS, Gibco) and transfected with 1 μg of plasmid once cells reached 90–100% confluency, using Mirus TransIT-LT1 (Mirus) or Lipofectamine 3000 (Invitrogen) according to manufacturer’s instructions. In brief, a mixture of plasmid DNA, Mirus TransIT-IL1 or Lipofectamine 3000 reagent, and Opti-MEM reduced serum medium (Gibco) was incubated according to manufacturer’s instructions with the following modifications: transfection mixture was incubated for 20 mins at room temperature, then added to cells in a drop-wise manner.

### In vitro HCV RNA transcription and electroporation

HCV genomic RNA was in vitro transcribed (MEGAscript T7 transcription kit; Thermo Fisher) from 1 μg of XbaI-digested HCV cDNA, including HJ3-5, JFH1-QL, E2-EMCV-p7, HJ3-5/^HA^p7, JFH1/QL/^HA^p7, or JFH1 replicon plasmids. The in vitro transcribed RNA was treated with DNase (Thermo Fisher) for 15 minutes at 37°C and then purified using an RNeasy RNA extraction kit (Qiagen, Valencia, CA). Purified RNA integrity and concentration were determined by agarose gel electrophoresis and NanoDrop Spectrophotometer (ThermoFisher). For electroporation, 10 μg of HCV RNA was mixed with 5 x 10^6^ SPCS1(-) or SPCS1(+) cells in cold 1X phosphate-buffered saline (PBS) in a 4 mm cuvette and then electroporated at 950 μF and 270 V with a Gene Pulser Xcell system (Bio-Rad, Hercules, CA). Electroporated cells were seeded into 12-well plates for HCV RNA analysis and 6-well plates for virus titration and HCV protein analysis.

## Reverse transcription and quantitative PCR (RT qPCR)

Intracellular HCV RNA was isolated by using an RNeasy RNA isolation kit (Qiagen, Valencia, CA). To quantitate the level of HCV RNA, a real-time RT-PCR assay was performed by using a QuantiNova Probe RT-PCR Kit (Qiagen, Valencia, CA) and a CFX96 real-time system (Bio-Rad, Hercules, CA) with custom-designed primer-probe sets (a forward primer: HCV84FP, 5’-GCCATGGCGTTAGTATGAGTGT-3’; a reverse primer: HCV 303RP, 5’-CGCCCTATCAGGCAGTACCACAA-3’; and a probe: HCV146BHQ, FAM-TCTGCGGAACCGGTGAGTACACC-DBH1).

### Virus titration

A 50 μl aliquot of serial 10-fold dilutions (1:10 to 1:1000) of cell culture supernatants (clarified by low-speed centrifugation) were inoculated onto naïve Huh-7.5 cells seeded 24 hr previously into 96-well plate at 5x10^4^ cells/well. Infected cells were maintained at 37°C in a 5% CO_2_ environment and supplemented with 100 μl of medium 24 hr later. The plate was then incubated for an additional 48 hr, then cells were fixed in methanol-acetone (1:1) at room temperature for 9 mins and then immunostained with monoclonal antibody to the Core protein (C7-50, 1:1000 dilution; Thermo Scientific, Rockford, IL), followed by washing (3X) and staining with fluorescein isothiocyanate-conjugated goat anti-mouse immunoglobulin G (IgG) antibody diluted 1:1000. Clusters of infected cells staining for Core antigen were considered to constitute a single infectious focus-forming unit (FFU). Virus titers (FFU/ml) were calculated from the sample dilutions, yielding 5 to 100 FFU per well.

### Western blot analysis

Cells were lysed at different time points post-electroporation with ice-cold lysis buffer [50 mM Tris-HCl (pH 7.5), 150 mM NaCl and 1% Triton X-100] and a complete protease inhibitor cocktail (GenDepot, Katy, TX). Aliquots of cell lysates containing 20 μg of proteins were separated by performing SDS-PAGE and transferred onto polyvinylidene difluoride (PVDF) membranes. Membranes were probed with monoclonal antibodies to E2 protein (Graciously provided by Dr. Michael Diamond at Washington University), Core protein (C7-50, 1:2000 dilution; Thermo Scientific, Rockford, IL), NS3 (9-G2, 1:2000 dilution; ViroGen, Watertown, MA), monoclonal anti-HA antibody (C29F4, 1:2000 dilution; Cell Signaling Technology, Danvers, MA), anti-Tubulin antibody (1:7,000 dilution; EMD Millipore, Billerica, MA) and anti-SPCS1 antibody (11847-1-AP, 1:2000 dilution, Proteintech). Proteins were visualized by subsequently probing the membranes with IRdye 800CW goat anti-mouse, and IRdye 680 goat anti-rabbit, and (Li-Cor Biosciences, Lincoln, NE), followed by imaging with an Odyssey infrared imaging system (Li-Cor Biosciences, Lincoln, NE).

### Endoglycosidase H treatment

Endoglycosidase H (Endo H; NEB, Hitchin, United Kingdom) treatment was performed according to the manufacturer’s recommendations. Briefly, 20 μg of protein was added to 3 μl of 10X Glycoprotein Denaturing Buffer then denatured at 100°C for 10mins. 3.5 μl of 10X GlycoBuffer 3 and 1.5 μl Endo H were added to samples and then these reaction mixtures were incubated for 2 hr at 37°C.

### Coimmunoprecipitation (Co-IP) assays

HEK293T cells were transfected with various plasmids and harvested for further assays 48 hr after transfection. Transfected cells were washed twice with phosphate-buffered saline (PBS) and scraped into lysis buffer (Cell Signaling Technology, Danvers, MA) with freshly added containing 1 X protease inhibitor (GenDepot, Katy, TX). After incubation for 30 min on ice, the lysate was centrifuged to remove the insoluble cell debris. Equal amounts of lysates were used for the Co-IP assay by incubating overnight with 0.5 μg of an anti-Flag antibody (COSMO BIO USA, INC. CA) at 4°C. After incubation, Dynabeads protein G (Invitrogen) 10 μl was added followed by incubation for 2 hr at room temperature. Wash Dynabeads with cold-PBS twice. The immune complexes were denatured by 37°C, 20 mins. Samples were then resolved by 14% SDS-PAGE and analyzed by Western blotting.

### Molecular dynamics (MD) simulations

The conformation of the p7 protein embedded in a membrane model was followed using MD simulations. Initially, three different p7 structures were tested: i) wt protein (ALENLVVLNAASVAGAHGILSFLVFFSAAWYIKGRLAPGAAYAFYGVWPL LLLLLALPPRAYA) (PDB ID: 2MTS, [48]); ii) mutant protein (obtained by replacing K33A and R35A on wt protein, PDB ID: 2MTS, [48]); iii) protein with HA-tag—YPYDVPDYAGGG (obtained by replacing the FLAG-tag with this HA-tag sequence, PDB ID: 3ZD0, [69]). To be consistent with the experimental data displayed in this study, four additional p7 structures were also tested: i) H77p7 model (ALENLVILNAASLAGTHGLVSFLVFFCFAWYLKGRWVPGAVYALYGMWPLLLLLLALPQRAYA), obtained by replacing different amino acid residues in the 2MTS structure; ii) JFH1p7 model (sequence: ALEKLVVLHAASAANCHGLLYFAIFFVAAWHIRGRVVPLTTYCLTGLWPFCLLLMALPRQAYA), also obtained by replacing different amino acid residues in 2MTS structure; iii) HA-H77p7 model (sequence: YPYDVPDYAGGGALENLVILNAASLAGTHGLVSFLVFFCFAWYLKGRWV PGAVYAFYGMWPLLLLLLALPQRAYA), obtained by replacing different amino acid residues in the 3ZD0 structure and iv) HA-JFH1p7 model (sequence: YPYDVPDYAGGGALEKLVVLHA ASAANCHGLLYFAIFFVAAWHIRGRVVPLTTYCLTGLWPFCLLLMALPRQAYA), also obtained by replacing different amino acid residues in the 3ZD0 structure. The starting structure of the p7 protein inserted in the membrane was constructed using the CHARMM-GUI web server [70]. The OPM database, [71] was used to obtain information on the protein orientation in the lipid bilayer. A bilayer composed of 40 POPC (1-palmitoyl-2-oleoyl-sn-glycero-3-phosphocholine) molecules in each leaflet was used as the model membrane. A rectangular simulation box was used, with 40 POPC lipids leaflet and a hydration thickness of 30 Å. K+ or Cl- ions were added to neutralize the protein and a final concentration of 0.1 mol∙dm^-3^ KCl was added to the system. Four different p7 structures were tested: i) wt protein (PDB ID code: 2MTS, [72]); ii) mutant protein (obtained by mutation of K33A and R35A on wt protein, PDB ID code: 2MTS, [72]); iii) protein with HA-tag—YPYDVPDYAGGG (obtained by mutation of the protein with the FLAG-tag, PDB ID code: 3ZD0, [52]). The p7 protein was described using the Amber ff99sb-ILDN forcefield, [73] and the lipid molecules with the LIPID17 forcefield from the AMBER project. The TIP3P model was used to describe the water molecules [74]. The simulations were conducted using GROMACS 2019 and the results were analyzed with GROMACS tools [75]. The system was first minimized and then equilibrated with a canonical ensemble for 1 ns, with an integration step of 1 fs, followed by 4 ns of successive isothermal-isobaric ensembles to gradually remove the position restraints from the protein and shift to an integration step of 2 fs. Equilibration was finished with 10ns of production without position restraints. The last structure of the equilibration was used as the initial structure for the subsequent studies of protein conformation. Fifty ns of the simulation were used to analyze the conformation of the helix-1, at the N-terminal of the p7 protein. Simulations were repeated at least once. The temperature was set to 310.15 K using the Berendsen thermostat for equilibration and the Nose-Hoover thermostat [76,77] with a coupling constant of 1.0 ps for production simulations. The pressure was set to 1.013 bar using the Berendsen barostat [78] for equilibration and the Parrinello-Rahman barostat [79] with a coupling constant of 5 ps for production simulations. Long-range electrostatic interactions were treated by the particle-mesh Ewald (PME) method [80,81] with a real space cut-off of 0.9 nm and Fourier spacing of 0.16 nm. The van der Waals interactions were treated with a cut-off of 0.9 nm. Long-range dispersion corrections to the pressure and energy were added [82]. Bonds were constrained using the LINCS algorithm [83] and the SETTLE algorithm [84] was used for the water molecules. Coordinates were saved every 5 ps. The center of mass (COM) motion (translational) of the protein-bilayer system relative to the solvent was removed every 10 steps of the simulation. Two COM groups were used: 1) membrane (lipids and protein), and 2) the solvent (water and ions). Simulations were visually analyzed with the VMD software (version 1.9.4.) [85].

### Statistical analysis

Unpaired Student’s t-test with Welch’s correction was performed by using GraphPad Prism version 9 software (GraphPad, La Jolla, CA) to determine the significance in differences between paired values from at least three independent experiments. A P value less than 0.05 was considered statistically significant.

## Supporting information

S1 Fig **The effect of SPCS1 trans-complementation in SPCS1(-) cells on H77- (A) or JFH1- (B) E2-p7 processing.** Two additional western blot results associated with Fig 1C data.
(TIF)

S2 Fig **The effect of SPCS1 trans-complementation in SPCS1(-) cells on H77- or JFH1- E2-p7 processing during HJ3-5 and JFH1 replication (A and B) and the effect of proteasome inhibitor (MG132) on E2 stability (B).** These additional western blot results are associated with Fig 4A data.
(TIF)

S3 Fig The effect of SPCS1 loss on JFH1 subgenomic RNA replication. **(A)** Organization of JFH1 subgenomic replicon. **(B)** Western blot detection of SPCS1 and JFH1 NS3 protein during JFH1 replicon RNA replication. **(C)** JFH1 replicon RNA replication determined by measuring HCV RNA levels at different time points post electroporation o JFH1 replicon RNA to SPCS1(+) or SPCS1(-) cells.
(TIF)

S4 Fig Interaction of SPCS1 with H77- or JFH1-NS2. The interaction between ^HA^SPCS1 and Flag-tagged H77-or JFH1-NS2 was determined by Flag co-immunoprecipitation (co-IP) assay following co-transfection of these plasmids to 293T cells.
(TIF)

S5 Fig Molecular dynamics simulations of H77 and JFH1 p7 and ^HA^p7 in the membrane environment. **(A and B)** Snapshots represent the position of the p7/TM1/helix-1 structure along the simulation time (50 ns) for H77 and JFH1 p7 structures using PDB ID: 2MTS template and H77 and JFH1 ^HA^p7 structures based PDB ID: 3ZD0 template. The p7/TM1/helix-1 and HA-tag helix are highlighted in red and green, respectively, and the positions of these helices at every 5 ns of the simulation time are depicted in translucent. The remaining region of the p7 protein is represented in grey. The membrane core is represented as a surface in white with the lipid heads represented as balls with phosphorous atoms in gold and oxygen in red. **(C)** Representation of the vector used to follow the angle distribution: the vector of the p7/TM1/helix-1 and the vector of the membrane is shown on the left. The angle distribution between the p7/TM1/helix-1 or HA-tag helix structures and the membrane normal along the simulation time is shown on the right. The different protein structures tested are represented with different colors; TM1/helix-1 of H77 p7 in black, JFH1 p7 in blue, and HA tag in H77 ^HA^p7 in green, and JFH1 ^HA^p7 in orange. The solid and dashed lines represent three different simulations, and data were smoothed using adjacent averaging with 200 points per window.
(TIF)
