# Supplementary figures and images for: SPCS1-Dependent E2-p7 processing determines HCV Assembly efficiency
Source: PLoS Pathog. 2022 Feb 7;18(2):e1010310. doi: 10.1371/journal.ppat.1010310 (PMC8853643; doi:10.1371/journal.ppat.1010310)

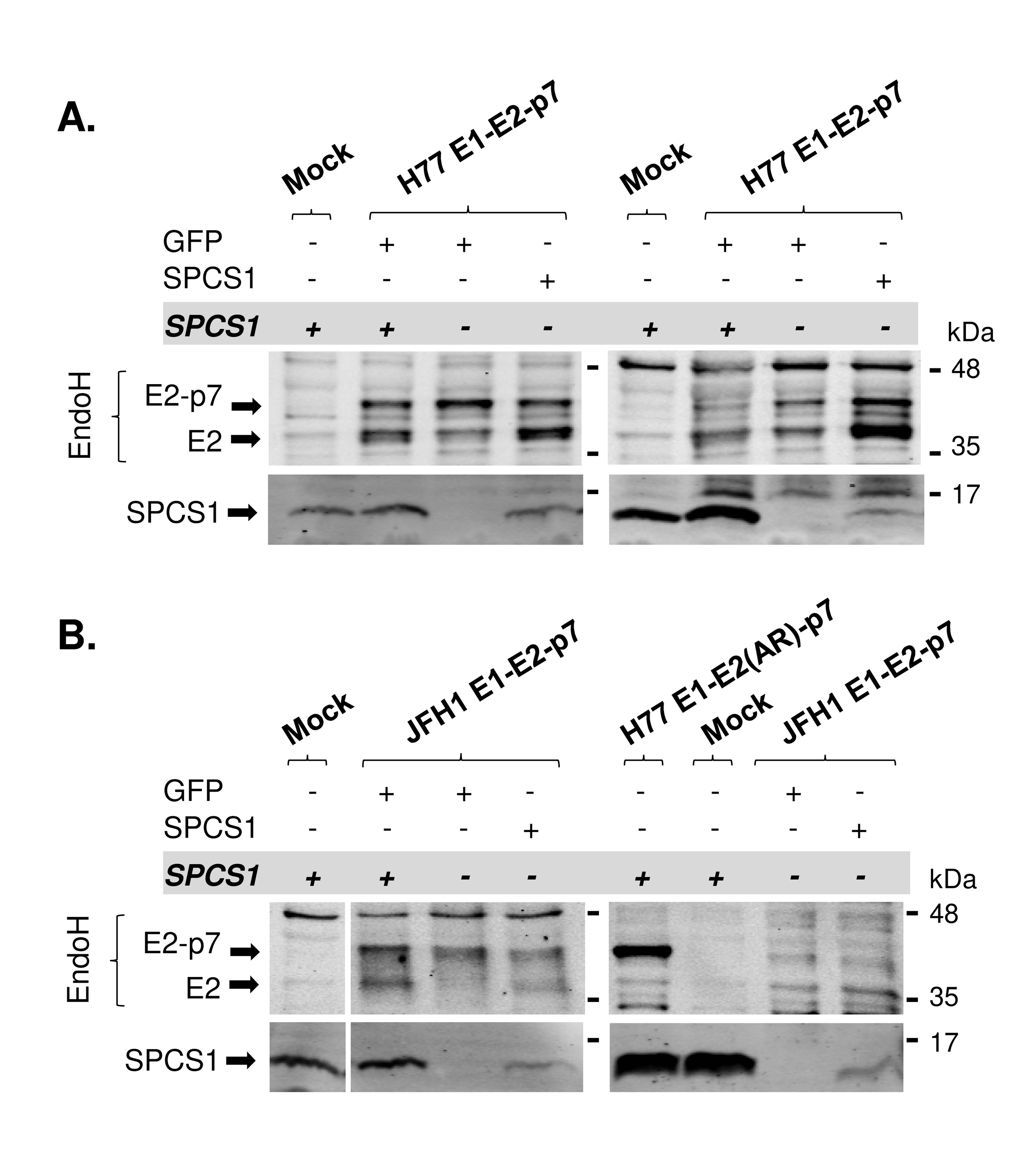

Supplement: S1 Fig — The effect of SPCS1 trans-complementation in SPCS1(-) cells on H77- (A) or JFH1- (B) E2-p7 processing. Two additional western blot results associated with Fig 1C data. (TIF) [file ppat.1010310.s001.tif]

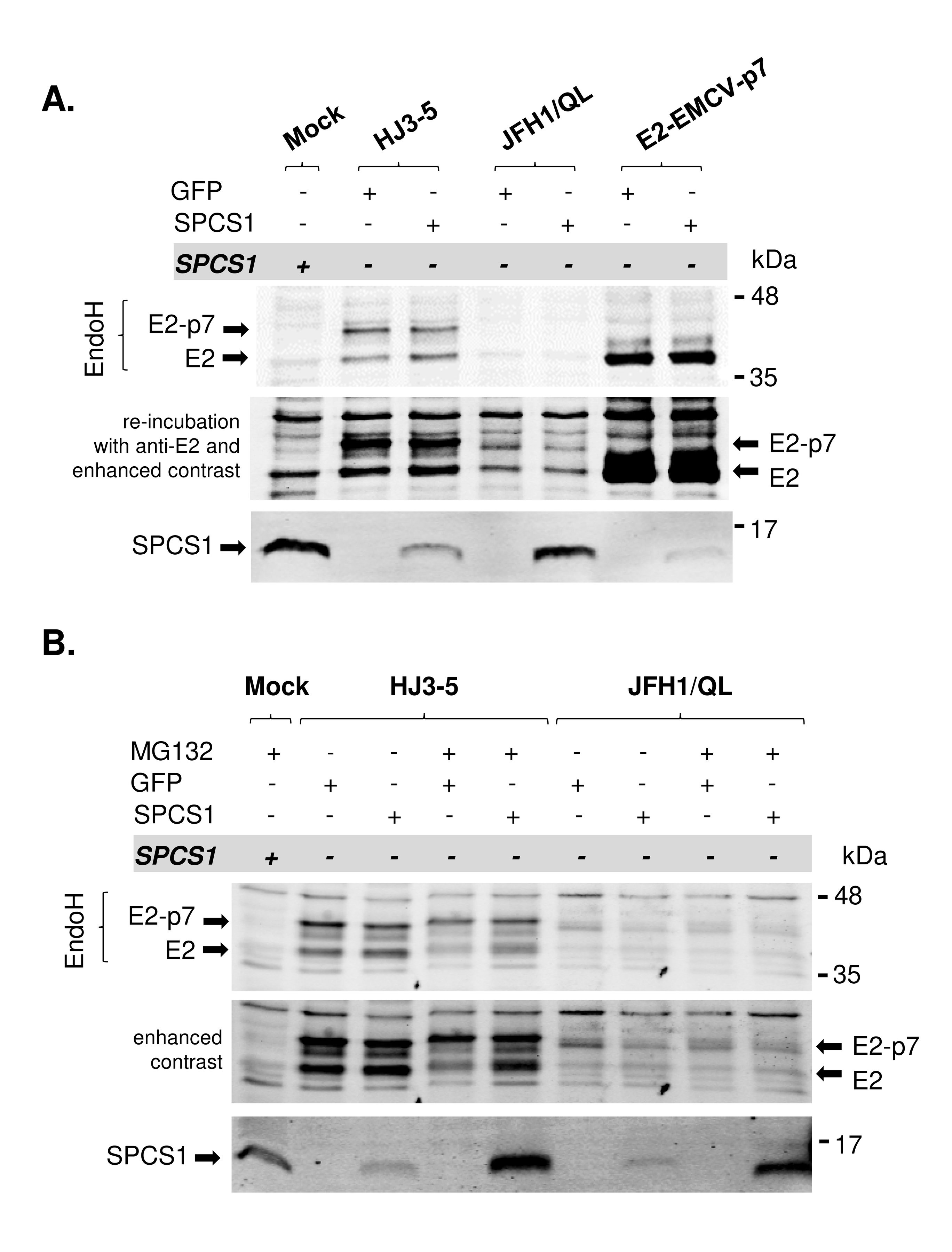

Supplement: S2 Fig — The effect of SPCS1 trans-complementation in SPCS1(-) cells on H77- or JFH1- E2-p7 processing during HJ3-5 and JFH1 replication (A and B) and the effect of proteasome inhibitor (MG132) on E2 stability (B). These additional western blot results are associated with Fig 4A data. (TIF) [file ppat.1010310.s002.tif]

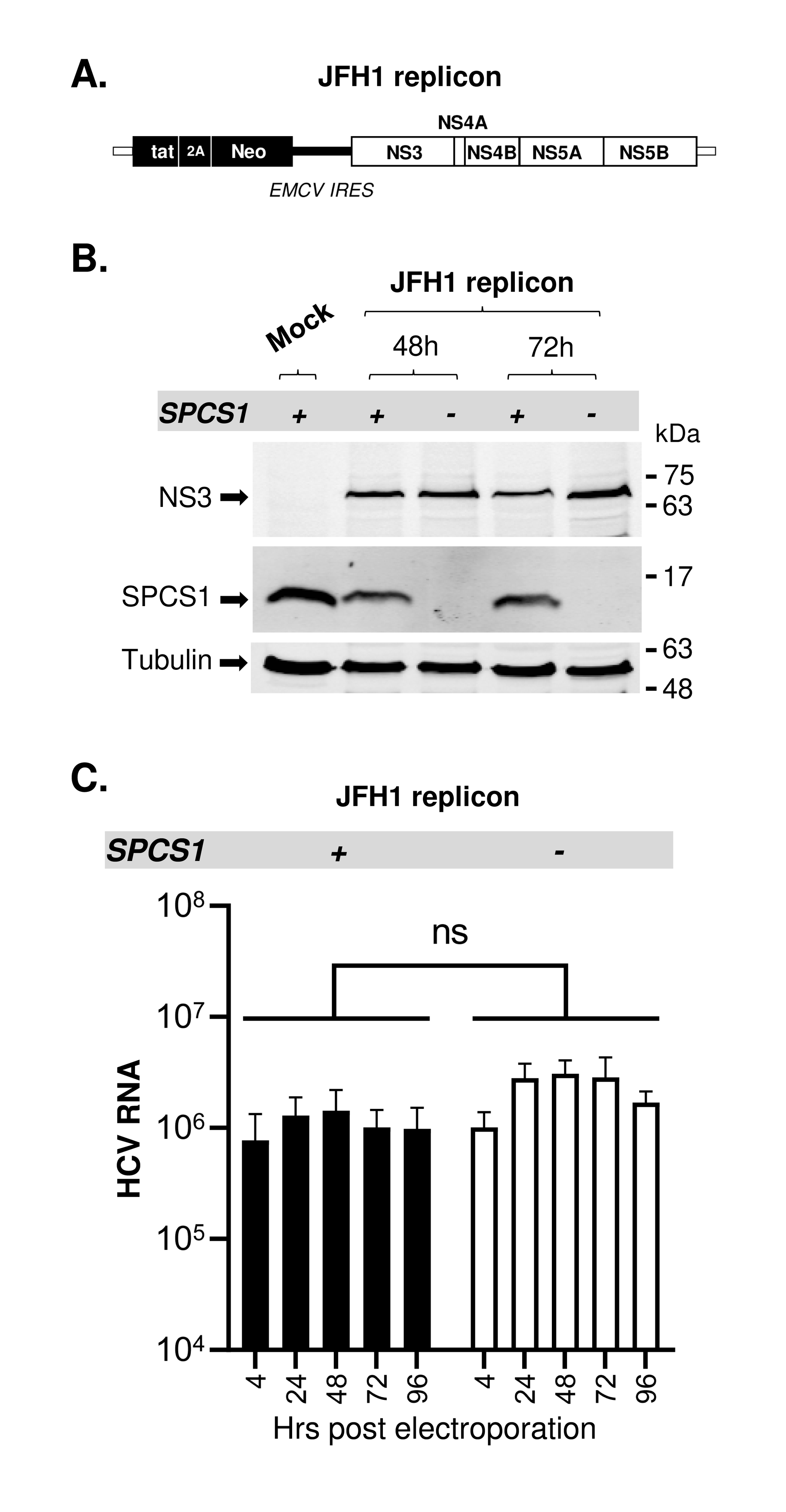

Supplement: S3 Fig — (A) Organization of JFH1 subgenomic replicon. (B) Western blot detection of SPCS1 and JFH1 NS3 protein during JFH1 replicon RNA replication. (C) JFH1 replicon RNA replication determined by measuring HCV RNA levels at different time points post electroporation o JFH1 replicon RNA to SPCS1(+) or SPCS1(-) cells. (TIF) [file ppat.1010310.s003.tif]

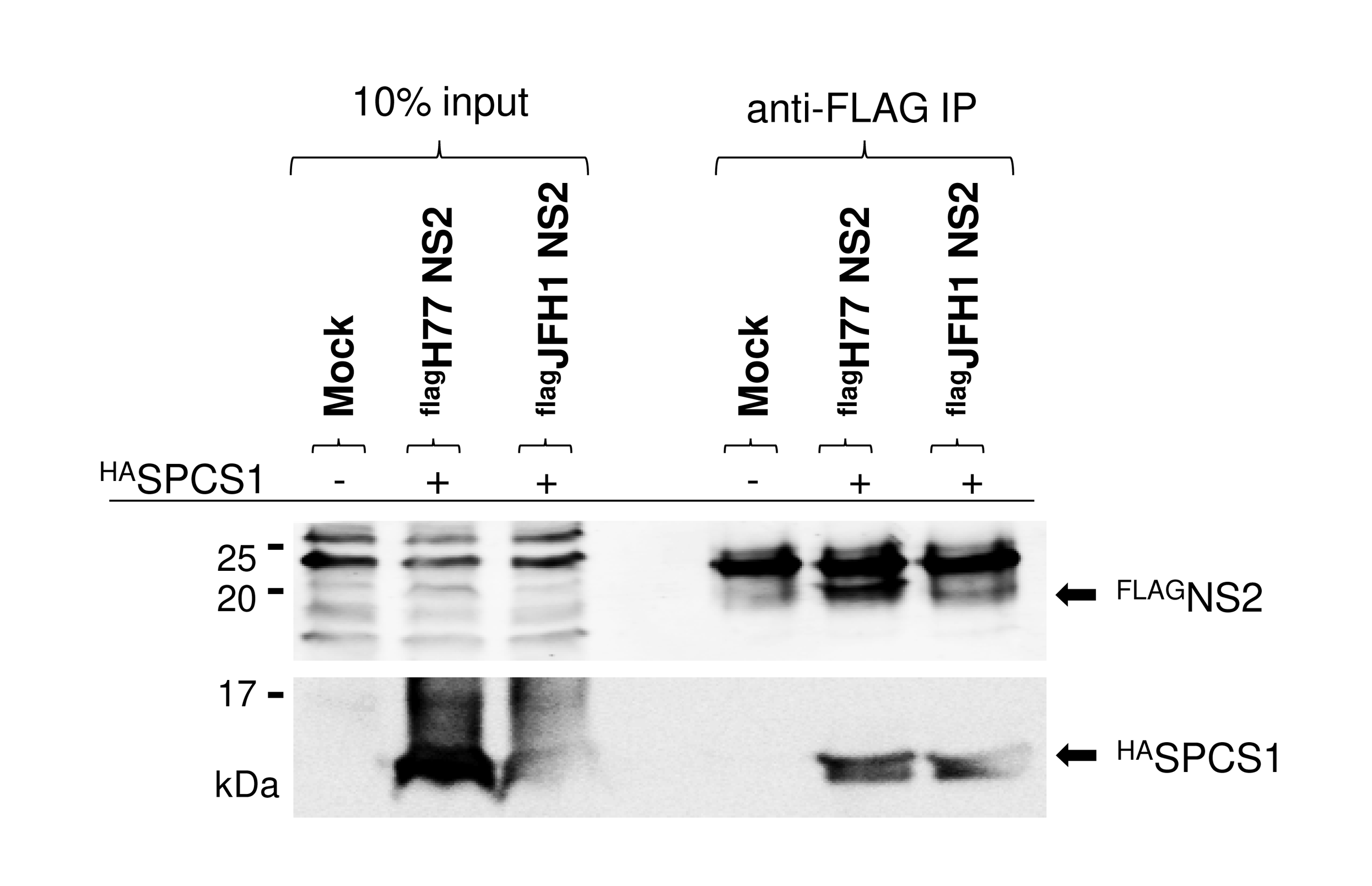

Supplement: S4 Fig — The interaction between HASPCS1 and Flag-tagged H77-or JFH1-NS2 was determined by Flag co-immunoprecipitation (co-IP) assay following co-transfection of these plasmids to 293T cells. (TIF) [file ppat.1010310.s004.tif]

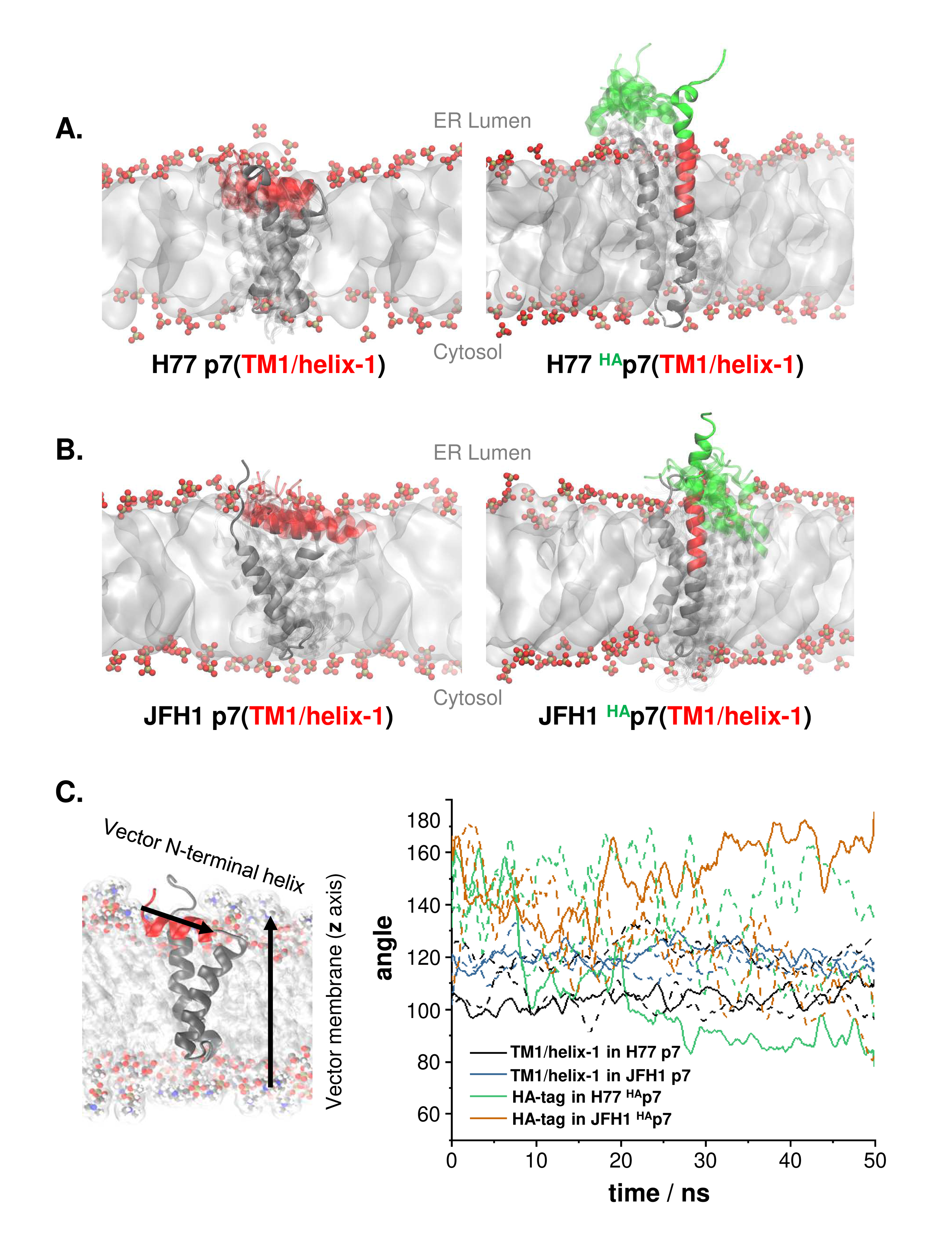

Supplement: S5 Fig — (A and B) Snapshots represent the position of the p7/TM1/helix-1 structure along the simulation time (50 ns) for H77 and JFH1 p7 structures using PDB ID: 2MTS template and H77 and JFH1 HAp7 structures based PDB ID: 3ZD0 template. The p7/TM1/helix-1 and HA-tag helix are highlighted in red and green, respectively, and the positions of these helices at every 5 ns of the simulation time are depicted in translucent. The remaining region of the p7 protein is represented in grey. The membrane core is represented as a surface in white with the lipid heads represented as balls with phosphorous atoms in gold and oxygen in red. (C) Representation of the vector used to follow the angle distribution: the vector of the p7/TM1/helix-1 and the vector of the membrane is shown on the left. The angle distribution between the p7/TM1/helix-1 or HA-tag helix structures and the membrane normal along the simulation time is shown on the right. The different protein structures tested are represented with different colors; TM1/helix-1 of H77 p7 in black, JFH1 p7 in blue, and HA tag in H77 HAp7 in green, and JFH1 HAp7 in orange. The solid and dashed lines represent three different simulations, and data were smoothed using adjacent averaging with 200 points per window. (TIF) [file ppat.1010310.s005.tif]
